# A New Extension of the Generalized Half Logistic Distribution with Applications to Real Data

**DOI:** 10.3390/e21040339

**Published:** 2019-03-28

**Authors:** Mustapha Muhammad, Lixia Liu

**Affiliations:** College of Mathematics and Information Sciences, Hebei Normal University, Shijiazhuang 050024, China; mmuhammad.mth@buk.ed.ng or

**Keywords:** generalized half logistic model, moments, maximum likelihood estimation, Shannon entropy, Renyi entropy, Kullback-Leibler divergence, stress-strength reliability analysis, 62E05, 62F10, 62F12

## Abstract

In this paper, we introduced a new three-parameter probability model called Poisson generalized half logistic (PoiGHL). The new model possesses an increasing, decreasing, unimodal and bathtub failure rates depending on the parameters. The relationship of PoiGHL with the exponentiated Weibull Poisson (EWP), Poisson exponentiated Erlang-truncated exponential (PEETE), and Poisson generalized Gompertz (PGG) model is discussed. We also characterized the PoiGHL sub model, i.e the half logistic Poisson (HLP), based on certain functions of a random variable by truncated moments. Several mathematical and statistical properties of the PoiGHL are investigated such as moments, mean deviations, Bonferroni and Lorenz curves, order statistics, Shannon and Renyi entropy, Kullback-Leibler divergence, moments of residual life, and probability weighted moments. Estimation of the model parameters was achieved by maximum likelihood technique and assessed by simulation studies. The stress-strength analysis was discussed in detail based on maximum likelihood estimation (MLE), we derived the asymptotic confidence interval of $R=P(X_{1}<X_{2})$ based on the MLEs, and examine by simulation studies. In three applications to real data set PoiGHL provided better fit and outperform some other popular distributions. In the stress-strength parameter estimation PoiGHL model illustrated as a reliable choice in reliability analysis as shown using two real data set.

## 1. Introduction

Most of the classical distributions used in the reliability studies are based on some certain assumptions and are not capable in accommodating non-monotone failure rates. Several attempts have been made to propose a new parametric model from the existing classical one in the last few decade. The advantage of these approaches for constructing a new probability model lies in the flexibility to model both monotonic and non-monotonic failure rate functions even though the existing distribution (or baseline) may have a monotonic failure rate. One of these techniques that receive significant attention is the convolution of the continuous and discrete probability model. For instance, the Poisson and exponential distribution were considered by [1] based on the minimum order statistics of random variables that follow the exponential distribution, and called the exponential Poisson (EP); the study of its properties, estimation, and application to earthquakes data was successfully achieved. In a similar way, [2] investigate the mixture of Burr XII and Poisson called Burr XII Poisson (BXIIP) some properties and superior performance over the Burr XII and some other models were demonstrated in an application to failure data. It can be seen that this technique allow us to propose more realistic statistical models that extend the well-known classical models and at the same time provide great flexibility in a variety of applications. The reader is referred to the following for an overview of the compound of discrete and continuous distribution: the exponential geometric (EG) [3], Poisson-exponential (PE) [4], generalized exponential-power series (GEPS) [5], linear failure rate-power series (LFPS) [6], exponentiated Weibull- Poisson (EWP) [7], exponentiated Weibull-logarithmic (EWL) [8], exponentiated Weibull power series (EWP) [9], complementary exponentiated BurrXII Poisson (CEBXIIP) [10], Poisson-odd generalized exponential (POGE) [11], half logistic Poisson (HLP) [12], Poison half logistic (PHL) [13] among others.

In this paper, we propose a new three-parameter extension of the generalized half logistic (GHL) distribution by employing additional parameter from zero-truncated Poisson distribution to the two-parameter GHL on the basis of the compounding technique. We call the new model Poisson-generalized half logistic (PoiGHL).

Some of the interest behind introducing the new PoiGHL distribution is to provide a new flexible parametric model for modeling complex data that arises in reliability studies, survival analysis, statistical mechanics, quality control, economics, biomedical studies, etc. The goal is to propose a new model that can accommodate an increasing, decreasing, unimodal, upside-down bathtub and bathtub curve failure rates for lifetime analysis, and provides many close form properties of the model with easy physical interpretations. In addition, to explore new mathematical expressions to utilized some mathematical ideas such as computational algorithms and techniques.

The paper is organized as follows, in Section 2, we derived the new model and some important mathematical and statistical properties. In Section 3, we discussed the Shannon and Renyi entropies and Kullback-Leibler divergence of the new distribution. In Section 4, characterization of the half logistic Poisson (HLP) by truncated moments is provided. In Section 5, the parameter estimation of the PoiGHL by maximum likelihood method is discussed and examine the estimators by simulation studies. In Section 6, the stress-strength reliability analysis of the PoiGHL ($R=P(X_{1}<X_{2})$) is discussed based on maximum likelihood estimation and assessed the estimators numerically by simulation studies. In Section 7, applications of the PoiGHL to real data sets are provided for illustration. Finally, conclusions in Section 8.

## 2. The PoiGHL Model and Properties

In this section, we derived the new model and investigate some of its important properties.

Among the parametric models, the half logistic distribution is perhaps one of the classical distribution widely used in statistical studies in several fields. The half logistic has a decreasing density and increasing failure rate. The density and hazard of HL are given by $t\left(y\right)=2\alpha e^{-\alpha y}{\left(1+e^{-\alpha y}\right)}^{-2}$, and $\tau \left(y\right)=\alpha {\left(1+e^{-\alpha y}\right)}^{-1}$ respectively. Therefore, it’s clear that the weakness of the HL distribution is the inability to accommodate non-monotone failure rates and unimodal density. Due to its wide application, the model has attracted several authors to propose its extension for added flexibility in modeling, for example, generalized half logistic (GHL) [14], power half logistic (PwHL) [15], Olapade-half logistic (OHL) [16] etc. Among them, we are interested in the two-parameters generalized half logistic distribution, some statistical studies and usefulness of the GHL can be found in [17].

The cumulative distribution and density functions of the generalized half logistic are defined by

(1) $$G\left(y\right)={\left(\frac{1-e^{-\alpha y}}{1+e^{-\alpha y}}\right)}^{a},\;\;\;\;\;y,\alpha ,a>0,$$

and

(2) $$g\left(y\right)=\frac{2a\alpha e^{-\alpha y}{\left(1-e^{-\alpha y}\right)}^{a-1}}{{\left(1+e^{-\alpha y}\right)}^{a+1}}\;\;\;\;\;y,\alpha ,a>0,$$

respectively.

Let $Y_{1},Y_{2},Y_{3},\cdots ,Y_{M}$ be a random sample of size *M* from the GHL distribution with cdf in (1), let *M* be a zero-truncated Poisson random variable independent of *Y* with probability mass function given by

(3) $$P\left(M=m\right)=\lambda^{m}{\left(\left(e^{\lambda }-1\right)m!\right)}^{-1},\;\lambda >0,\;m\in N.$$

Let $X=min{\left\{Y_{i}\right\}}_{i=1}^{m}$, then the conditional random variable X|M=m has cdf

$$\begin{array}{cc} F(X|M=m) & =1-P\left(X>x|M\right)=1-P\left(Y_{1}>x,Y_{2}>x,\cdots ,Y_{m}>x\right)\\ & =1-P^{m}\left(Y_{1}>x\right)=1-{\left[1-P\left(Y_{1}\leq x\right)\right]}^{m}\\ & =1-{\left[1-F\left(x\right)\right]}^{m}\\ & =1-{\left[1-{\left(\frac{1-e^{-\alpha x}}{1+e^{-\alpha x}}\right)}^{a}\right]}^{m}. \end{array}$$

The cumulative distribution function of PoiGHL(α,λ,a) is the unconditional cdf F(x;α,λ,a) of *X* and obtained as

(4) $$\begin{array}{cc} F_{PoiGHL}\left(x\right) & =\sum_{m=0}^{\infty }F\left(X|M=m\right)P\left(M=m\right)\\ & =\frac{1-e^{-\lambda {\left(\frac{1-e^{-\alpha x}}{1+e^{-\alpha x}}\right)}^{a}}}{1-e^{-\lambda }},\;\;\;\;x,\alpha ,\;\lambda ,a>0. \end{array}$$

The corresponding density function f(x;α,λ,a) is given by

(5) $$f_{PoiGHL}\left(x\right)=\frac{2\;a\;\alpha \;\lambda \;e^{-\alpha x}\;{\left(1-e^{-\alpha x}\right)}^{a-1}}{\left(1-e^{-\lambda }\right)\;{\left(1+e^{-\alpha x}\right)}^{a+1}}e^{-\lambda {\left(\frac{1-e^{-\alpha x}}{1+e^{-\alpha x}}\right)}^{a}}\;\;\;\;\;x,\alpha ,\lambda ,a>0.$$

The survival and failure rate functions (hrf) of the PoiGHL are given by

(6) $$S_{PoiGHL}\left(x\right)=\frac{e^{-\lambda {\left(\frac{1-e^{-\alpha x}}{1+e^{-\alpha x}}\right)}^{a}}-e^{-\lambda }}{1-e^{-\lambda }},$$

and

(7) $$h_{PoiGHL}\left(x\right)=\frac{2a\;\alpha \;\lambda \;e^{-\alpha x}\;{\left(1-e^{-\alpha x}\right)}^{a-1}\;e^{-\lambda {\left(\frac{1-e^{-\alpha x}}{1+e^{-\alpha x}}\right)}^{a}}}{\left(e^{-\lambda {\left(\frac{1-e^{-\alpha x}}{1+e^{-\alpha x}}\right)}^{a}}-e^{-\lambda }\right)\;{\left(1+e^{-\alpha x}\right)}^{a+1}}\;,$$

respectively, where x,α,λ,a>0.

**Interpretation** **1.** *Let Z be a random variable with pdf of the form $c\left(z\right)=\frac{\lambda e^{-\lambda z}}{1-e^{-z}}$ for z∈(0,1), and λ∈R. Let G(y) be a valid cumulative distribution function of an absolutely continuous random variable Y. A family of generalized cumulative distribution function of Y can be generated from the integral*

(8) $$F\left(x\right)=\int_{0}^{G(x)}\frac{\lambda e^{-\lambda z}}{1-e^{-\lambda }}dz=\frac{1-e^{-\lambda G(x)}}{1-e^{-\lambda }},$$

*therefore, the cumulative distribution given in (4) can be a special case of (8) by taking the cdf of the GHL, $G\left(x\right)={\left(\frac{1-e^{-\alpha x}}{1+e^{-\alpha x}}\right)}^{a}$.*

Notice that (8) can be used to generate several new cdf by taking various form of baseline cdf G(x).

**Interpretation** **2.** *Let (X,W) be a random vector with joint density function f(x,w) defined on $R^{2}$. Suppose that the conditional cumulative distribution of X given W=w is H(x|w) and W∼c(w). Then the following defines the unconditional survival function of X*

(9) $$s\left(x\right)=\int \bar{H}\left(x|w\right)c\left(w\right)dw.$$

*The survival function s(x) is obtained by compounding the survival function $\bar{H}\left(x|w\right)=1-H\left(x|w\right)$ and the density of c(w). Suppose that the survival function*

$$\bar{H}\left(x|w\right)=e^{-w\left(\frac{1-e^{-\lambda \tau (x;\alpha ,a)}}{e^{-\lambda \tau (x;\alpha ,a)}-e^{-\lambda }}\right)}$$

*where $\tau \left(x;\alpha ,a\right)={\left(\frac{1-e^{-\alpha x}}{1+e^{-\alpha x}}\right)}^{a}$, x,α,λ,a>0, and W assumed to have exponential distribution with mean 1, then X has survival function in (6).*

**Proof.**  For all x,w,α,λ,a>0, the survival function is given as

$$\begin{array}{cc} s(x) & =\int \bar{H}\left(x|w\right)c\left(w\right)dw.\\ & =\int_{0}^{\infty }e^{-w\left(\frac{1-e^{-\lambda \tau (x;\alpha ,a)}}{e^{-\lambda \tau (x;\alpha ,a)}-e^{-\lambda }}\right)}e^{-w}dw=\int_{0}^{\infty }e^{-w\left(\frac{1-e^{-\lambda \tau (x;\alpha ,a)}}{e^{-\lambda \tau (x;\alpha ,a)}-e^{-\lambda }}+1\right)}dw\\ & ={\left(\frac{1-e^{-\lambda \tau (x;\alpha ,a)}}{e^{-\lambda \tau (x;\alpha ,a)}-e^{-\lambda }}+1\right)}^{-1}=\frac{e^{-\lambda {\left(\frac{1-e^{-\alpha x}}{1+e^{-\alpha x}}\right)}^{a}}-e^{-\lambda }}{1-e^{-\lambda }}. \end{array}$$

☐

**Proposition** **1.** *The limiting distribution of PoiGHL(α,λ,a) as $\lambda \to 0^{+}$ is the GHL(α,a).*

$$\lim_{\lambda \to 0^{+}}F_{PoiGHL}\left(x\right)={\left(\frac{1-e^{-\alpha x}}{1+e^{-\alpha x}}\right)}^{a}.$$

**Proof.**  Straight forward. ☐

**Proposition** **2.** *The pdf of PoiGHL is decreasing corresponding to $\psi =\{{\left(\alpha ,\lambda ,a\right)}^{T}|\alpha >0,\lambda >2,a\in \left(\frac{2}{\lambda },1\right)\}$, where $\psi \in R_{+}^{3}$ is the parameter region.*

**Proof.**  We show that dlogf(x)/dx is negative.

(10) $$\frac{d\log f(x)}{dx}=-\alpha +\frac{2\alpha e^{-\alpha x}}{1+e^{-\alpha x}}+\frac{\alpha \left(a-1\right)e^{-\alpha x}}{1-e^{-\alpha x}}+\frac{\alpha \left(a-1\right)e^{-\alpha x}}{1+e^{-\alpha x}}-\frac{2a\alpha \lambda \;e^{-\alpha x}}{{\left(1+e^{-\alpha x}\right)}^{2}}{\left(\frac{1-e^{-\alpha x}}{1+e^{-\alpha x}}\right)}^{a-1},$$

from (10), for a<1, define $\pi \left(x\right)=\frac{2\alpha e^{-\alpha x}}{1+e^{-\alpha x}}\left(1-\frac{a\;\lambda }{\left(1+e^{-\alpha x}\right)}{\left(\frac{1-e^{-\alpha x}}{1+e^{-\alpha x}}\right)}^{a-1}\right)$. If a<1 and π(x)<0, the (10) is negative. notice that, ${\left(\frac{1-e^{-\alpha x}}{1+e^{-\alpha x}}\right)}^{a-1}\geq 1$ for a<1, so, π is negative if $\frac{a\;\lambda }{\left(1+e^{-\alpha x}\right)}>1$, therefore, we need $a\;\lambda >max\left(1+e^{-\alpha x}\right)=2$, implies that $a>\frac{2}{\lambda }$, but a<1 therefore, we must have λ>2. ☐

The limiting behavior of the density in (5) and hazard function in (7) are: if x→0, then f(x)∼0 for a>1, f(x)∼∞ for a<1, $f\left(x\right)∼\frac{\alpha \lambda }{2(1-e^{-\lambda })}$ for a=1, and if x→∞, then f(x)∼0 ∀a>0. If x→0, then h(x)∼0 for a>1, h(x)∼∞ for a<1, $h\left(x\right)∼\frac{\alpha \lambda }{2(1-e^{-\lambda })}$ for a=1.

**Figure 1:**
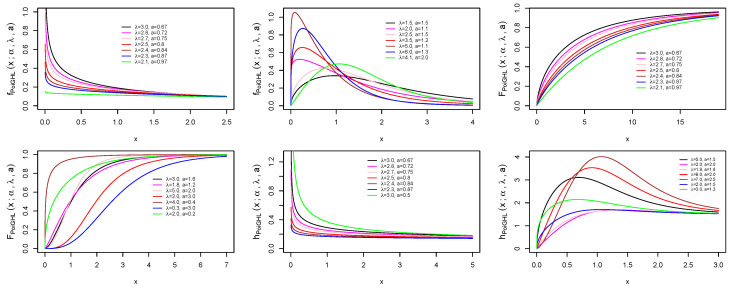
Plots of the pdf, cdf and hrf of the PoiGHL for various parameter values: f(x;0.1,λ,a) (**top left**) and f(x;0.6,λ,a) (**top middle**), F(x;0.1,λ,a) (**top right**) and F(x;0.8,λ,a) (**bottom left**), h(x;0.1,λ,a) (**bottom middle**) and h(x;1.5,λ,a) (**bottom right**).

**Figure 2:**
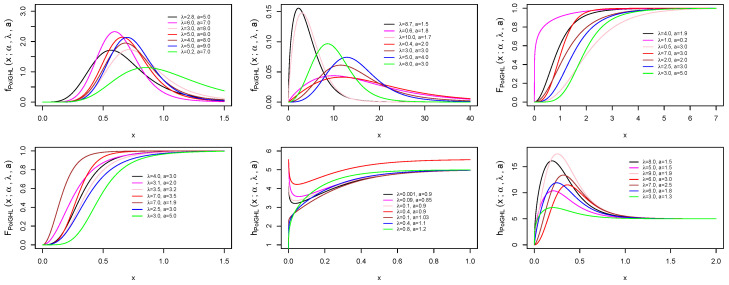
Plots of the pdf, cdf and hrf of the PoiGHL for various parameter values: f(x;3.0,λ,a) (**top left**) and f(x;0.1,λ,a) (**top middle**), F(x;1.0,λ,a) (**top right**) and F(x;4.0,λ,a) (**bottom left**), h(x;5.0,λ,a) (**bottom middle**) and h(x;5.0,λ,a) (**bottom right**).

Figure 1 and Figure 2 illustrate the plots of the pdf, cdf and hrf of PoiGHL for some parameter values. Clearly from the figure, the hrf of PoiGHL can accommodate failure rate with decreasing, increasing, bathtub-shaped, upside down bathtube-shaped (or unimodal) and increasing-decreasing-constant.

### 2.1. Quantiles

Here, we present the quantile of PoiGHL and some applications. The quantile function of a distribution has many applications in both theoretical and applied statistics, such as a means for estimation of model parameter, generating random data, study of skewness and kurtosis, and in computations of some properties of a distribution etc. The quantile of PoiGHL is derived as

(11) $$\xi \left(p\right)=\alpha^{-1}\left[\ln\left(1+W_{\lambda }^{1/a}\left(p\right)\right)-\ln\left(1-W_{\lambda }^{1/a}\left(p\right)\right)\right],$$

where $W_{\lambda }\left(p\right)=\left(\frac{\log\left(1-p\left(1-e^{-\lambda }\right)\right)}{-\lambda }\right)$ and 0<p<1. The Median of PoiGHL is ξ(0.5), Table 1 shows that, as the α,λ,a increases, the median is increasing-decreasing-increasing- decreasing. The algorithm for generating random data that follow PoiGHL is: let P∼U(0,1) then, $X=\alpha^{-1}\left[\ln\left(1+W_{\lambda }^{1/a}\left(P\right)\right)-\ln\left(1-W_{\lambda }^{1/a}\left(P\right)\right)\right]$ is a random variable following PoiGHL, and U(0,1) is a uniform distribution.

Bowley’s skewness (BS) [18] and Moor’s kurtosis (MK) [19] are important tools used to investigate the skewness and kurtosis of a distribution. BS and MK are defined as functions of ξ(.) in (11) by

$$BS=\frac{\xi \left(\frac{3}{4}\right)-2\xi \left(\frac{2}{4}\right)+\xi \left(\frac{1}{4}\right)}{\xi \left(\frac{3}{4}\right)-\xi \left(\frac{1}{4}\right)},\;and\;MK=\frac{\xi \left(\frac{7}{8}\right)-\xi \left(\frac{5}{8}\right)+\xi \left(\frac{3}{8}\right)-\xi \left(\frac{1}{8}\right)}{\xi \left(\frac{6}{8}\right)-\xi \left(\frac{2}{8}\right)}.$$

**Figure 3:**
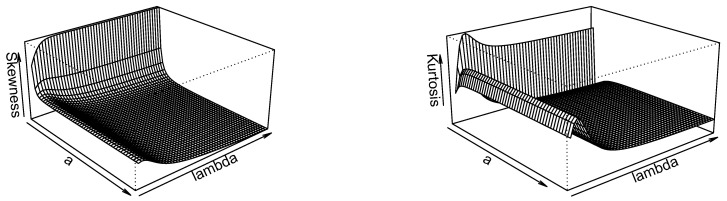
Plots of the Bowley’s skewness (BS) and Moor’s kurtosis (MK) of PoiGHL for α=1.

Figure 3 shows that the skewness is decreasing in *a* and increasing then decreasing in λ while the kurtosis is increasing in *a* and increasing then decreasing in λ.

#### Quantile Series Expansion

The power series representations of the quantile function can be used to compute some useful properties of PoiGHL such as moments, etc. Using the power series of $\log\left(1-u\right)=-\sum_{i=1}^{\infty }\frac{u^{i}}{i}$ and $\log\left(1+u\right)=\sum_{i=1}^{\infty }{\left(-1\right)}^{i+1}\frac{u^{i}}{i}$ for |u|<1, from the Equation (11) we can write

(12) $$\left[\ln\left(1+W_{\lambda }^{1/a}\left(p\right)\right)-\ln\left(1-W_{\lambda }^{1/a}\left(p\right)\right)\right]=\sum_{i=1}^{\infty }b_{i}W_{\lambda }^{i/a}\left(p\right)$$

where $b_{i}=\left[1-{\left(-1\right)}^{i}\right]/i$ and by the expansion of $W_{\lambda }^{i/a}\left(p\right)=\lambda^{-\frac{i}{a}}{\left[\sum_{j=1}^{\infty }\frac{p^{j}{\left(1-e^{-\lambda }\right)}^{j}}{j}\right]}^{i/a},$ therefore (11) is

(13) $$\xi \left(p\right)=\alpha^{-1}\sum_{i=1}^{\infty }b_{i}^{*}{\left[\sum_{j=1}^{\infty }c_{j}p^{j}\right]}^{i/a}$$

where $b_{i}^{*}=b_{i}\lambda^{-\frac{i}{a}}$ and $c_{j}={\left(1-e^{-\lambda }\right)}^{j}/j$. Now, we can applying the Taylor series expansion of $u^{v}$ defined by $u^{v}=\sum_{k=0}^{\infty }\frac{{\left(v\right)}_{k}}{k!}{\left(u-1\right)}^{k}$, where ${\left(v\right)}_{k}$ is the descending factorial with ${\left(v\right)}_{0}=1$ and ${\left(v\right)}_{k}=v\left(v-1\right)\cdots \left(v-k+1\right),\;k\geq 1$, then, if we let $c_{0}^{*}=-1$ and $c_{j}^{*}=c_{j},\;j\geq 1$, then we can write Equation (13) as

(14) $$\xi \left(p\right)=\alpha^{-1}\sum_{i=1}^{\infty }b_{i}^{*}\left[\sum_{k=0}^{\infty }\frac{{\left(i/a\right)}_{k}}{k!}{\left(\sum_{j=0}^{\infty }c_{j}^{*}p^{j}\right)}^{k}\right].$$

From [20], the expansion of power series to the power of k≥1, k∈N, we get

(15) $${\left(\sum_{j=0}^{\infty }c_{j}^{*}p^{j}\right)}^{k}=\sum_{j=0}^{\infty }d_{j}p^{j}$$

where $d_{0}={\left(c_{0}^{*}\right)}^{k}$ and $d_{j}=\frac{1}{md_{0}}\sum_{j=1}^{m}\left[jk-m+j\right]c_{j}^{*}d_{m-j}$ for m≥1 therefore, by (15) in (14),

$$\xi \left(p\right)=\sum_{i=1}^{\infty }\sum_{k,j=0}^{\infty }e_{i,j,k}p^{j},$$

where $e_{i,j,k}=\frac{b_{i}^{*}{\left(i/a\right)}_{k}d_{j}}{\alpha k!}$.

### 2.2. Moments, Mean Deviations, Bonferroni and Lorenz Curves

Most of the important features and characteristics of distribution are studied through its moments such as skewness, kurtosis, variation, dispersion, etc. In this subsection, we compute the *r*th moments, moment generating function, incomplete moments, mean deviations, Bonferonni, and Lorenz curves, we also investigate them numerically.

#### 2.2.1. Moments

The moments of the PoiGHL distribution is computed as follows.

**Theorem** **1.** *The rth moments of PoiGHL(α,λ,a) is given by*

(16) $$\mu_{r}=C_{i,j}\;B_{0r}\left(a\left(i+1\right),j+1\right)\;\;\;\;\;\;\;\;r\in N,$$

*where Ci,j=∑j=0∞wi(−1)rαr−(a(i+1)+1)j and $w_{i}=\sum_{i=0}^{\infty }\frac{{\left(-1\right)}^{i}2\;a\alpha \lambda^{i+1}}{i!\;(1-e^{-\lambda })}$.*

**Proof.**  $$\mu_{r}=E\left(X^{r}\right)=\int_{0}^{\infty }x^{r}f\left(x\right)dx.$$

By the expansion of $e^{-\lambda {\left(\frac{1-e^{-\alpha x}}{1+e^{-\alpha x}}\right)}^{a}}$ and letting $u=1-e^{-\alpha x}$ we have

$$\begin{array}{cc} \mu_{r} & =w_{i}\int_{0}^{\infty }\frac{x^{r}e^{-\alpha x}{\left(1-e^{-\alpha x}\right)}^{a(i+1)-1}}{{\left(1+e^{-\alpha x}\right)}^{a(i+1)+1}}dx,\\ & =\frac{w_{i}{\left(-1\right)}^{r}}{\alpha^{r+1}}\int_{0}^{1}\frac{\ln^{r}\left(1-u\right)u^{a(i+1)-1}}{{\left(1+\left(1-u\right)\right)}^{a(i+1)+1}}du, \end{array}$$

where $w_{i}=\sum_{i=0}^{\infty }\frac{{\left(-1\right)}^{i}2\;a\alpha \lambda^{i+1}}{i!\;(1-e^{-\lambda })}$. Applying the generalized binomial expansion in ${\left(1+\left(1-u\right)\right)}^{-(a(i+1)+1)}$, we get.

$$\mu_{r}=C_{i,j}\int_{0}^{1}\ln^{r}\left(1-u\right)\;{\left(1-u\right)}^{j}\;u^{a(i+1)-1}du,$$

where $C_{i,j}=\sum_{j=0}^{\infty }\frac{w_{i}{\left(-1\right)}^{r}\Gamma \left(a\left(i+1\right)+j+1\right)}{\alpha^{r+1}j!\Gamma \left(a\left(i+1\right)+1\right)}$. The integral part above is *r*th partial derivative of beta function, thus,

$$\mu_{r}=C_{i,j}\;B_{0r}\left(a\left(i+1\right),j+1\right),$$

where $B_{kr}\left(w,b\right)=\frac{\partial^{k+r}}{\partial w^{k}\partial b^{r}}B\left(w,b\right)$. ☐

The moment generating function $M_{X}\left(t\right)$ of PoiGHL can be derived using the expansion of $e^{tx}=\sum_{r=0}^{\infty }\left(t^{r}/r!\right)x^{r}$ and Equation (16) as.

$$M_{X}\left(t\right)=\sum_{r=0}^{\infty }\frac{C_{i,j}t^{r}}{r!}\;B_{0r}\left(a\left(i+1\right),j+1\right).$$

Moreover, the central moments $\mu_{r}$ in (16) can be used to compute the higher order moments by taking r=1,2,3⋯. Then the variance ($\sigma^{2}$), coefficient of variation (CV), skewness ($\gamma^{3}$) and kurtosis ($\gamma^{4}$) of the PoiGHL could be obtain from $\sigma^{2}=\mu_{2}-\mu_{1}^{2}$, $CV=\sqrt{\frac{\mu_{2}}{\mu_{1}^{2}}-1}$, $\gamma^{3}=\left(\mu_{3}-3\mu_{2}\mu_{1}+2\mu_{1}^{3}\right)/{\left(\mu_{2}-\mu_{1}^{2}\right)}^{3/2}$, $\gamma^{4}=\left(\mu_{4}-4\mu_{3}\mu_{1}+6\mu_{2}\mu_{1}^{2}-3\mu_{1}^{4}\right)/{\left(\mu_{2}-\mu_{1}^{2}\right)}^{2}$. Table 1 described that, as the α,λ,a increases, the first six moments, variance, coefficient of variation decreases, while the skewness and kurtosis are decreasing -increasing- decreasing.

It is also of interest to compute the conditional moments of PoiGHL. The main application of the incomplete moment refers to the mean deviations, mean residual lifetime functions, Bonferroni and Lorenz curves. The lower incomplete moment $\psi_{r}\left(t\right)=\int_{0}^{t}x^{r}f\left(x\right)dx$ of PoiGHL is obtained by considering (16). The resulting integral is infinite series of derivative incomplete beta function as

(17) $$\psi_{r}\left(t\right)=\sum_{r=0}^{\infty }C_{i,j}\;\frac{\partial^{r}}{\partial v^{r}}B\left(\varphi (t);a\left(i+1\right),j+1\right),$$

where $\varphi \left(t\right)=1-e^{-\lambda t}$, v=j+1, and $C_{i,j}$ is given in Proposition 1. The upper incomplete moment can be derived from (16) and (17).

#### 2.2.2. Mean Deviations, Bonferroni and Lorenz Curves

Now, we compute the mean deviation about the mean ($md_{1}\left(x\right)$) and the mean deviation about the median ($md_{2}\left(x\right)$). If *X* has the PoiGHL, then we can derive the mean deviations about the mean $\mu_{1}=E\left(X\right)$ and the mean deviations about the median *M* by

$$\begin{array}{cc} md_{1}\left(x\right) & =\int_{0}^{\infty }|x-\mu |f\left(x\right)dx=2\left[\mu F(\mu )-J(\mu )\right],\\ md_{2}\left(x\right) & =\int_{0}^{\infty }|x-M|f\left(x\right)dx=\mu -2J\left(M\right). \end{array}$$

The measures $md_{1}\left(x\right)$ and $md_{2}\left(x\right)$ can be calculated using the relationship J(.). By considering (17) we have

(18) $$J\left(d\right)=\int_{0}^{d}xf\left(x\right)dx=\sum_{r=0}^{\infty }C_{i,j}\;\frac{\partial }{\partial v}B\left(\varphi (d);a\left(i+1\right),j+1\right),$$

where $\varphi \left(d\right)=1-e^{-\lambda d}$. Table 1 indicated that $md_{1}\left(x\right)$ and $md_{2}\left(x\right)$ decreases as α,λ,a increases.

**Figure 4:**
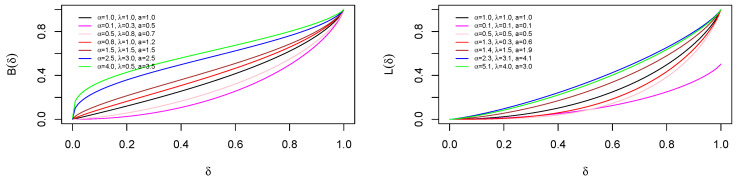
Plots of the Bonferroni and Lorenz curves for various parameter values.

The Bonferroni and Lorenz curves are income inequality measures that are also applicable to other areas including economics, demography, and insurance. Here, the Bonferroni and Lorenz curves can be computed by using (18). For a given probability δ, the Bonferroni curve of PoiGHL is given by

$$B\left(\delta \right)=\frac{1}{\mu \;\delta }\int_{0}^{q}xf\left(x\right)dx=\frac{J(q)}{\mu \;\delta }=\sum_{r=0}^{\infty }\frac{C_{i,j}}{\mu \;\delta }\;\frac{\partial }{\partial v}B\left(\varphi (q);a\left(i+1\right),j+1\right),$$

and the Lorenz curve is

$$L\left(\delta \right)=\frac{1}{\mu }\int_{0}^{q}xf\left(x\right)dx=\frac{J(q)}{\mu }=\sum_{r=0}^{\infty }\frac{C_{i,j}}{\mu }\;\frac{\partial }{\partial v}B\left(\varphi (q);a\left(i+1\right),j+1\right),$$

where *q* is the quantile at δ which can be derived from (11), hence $\varphi \left(q\right)=1-e^{-\frac{\lambda }{\alpha }\left(\ln\left(1+W_{\lambda }^{1/a}\left(\delta \right)\right)-\ln\left(1-W_{\lambda }^{1/a}\left(\delta \right)\right)\right)}$, and $W_{\lambda }\left(\delta \right)=\left(\frac{\log\left(1-\delta \left(1-e^{-\lambda }\right)\right)}{-\lambda }\right)$. Figure 4 show the plots of the Bonferroni and Lorenz curves for various parameter values.

### 2.3. Moments of Residual Life

Given that there was no failure of a component prior to the time t, the residual life $\left(M_{w}\left(t\right)\right)$ of a component is the period beyond the time *t* until failure. It is defined by the conditional moment of a random variable X−t|X>t. The reversed residual life $\left({\tilde{M}}_{w}\left(t\right)\right)$ of a component is the conditional moment of a random variable t−X|X≤t which describes the time elapsed from the failure of a component given that its life is less than or equal to t. These two measures play a vital role in survival analysis and life testing. In this subsection, we are interested to present an explicit form of the *w*th moment for both of them.

**Proposition** **3.** *Let t≥0 and w∈N, then, the wth moments of residual life of PoiGHL distribution is given by*

Mw(t)=1S(t)∑r=0wwr(−t)w−rμr−∑r=0∞Ci,j∂r∂vrB(φ(t);a(i+1),j+1),

*where S(.)=1−F(.) is the survival function of PoiGHL, $\varphi \left(t\right)=1-e^{-\lambda t}$, v=j+1, and $C_{i,j}$ is given in Proposition 1.*

**Proof.**  Mw(t)=E[(X−t)w|X>t]=1S(t)∫t∞(x−t)wf(x)dx=1S(t)∑r=0wwr(−t)w−r∫t∞xrf(x)dx

☐

**Proposition** **4.** *Let t≥0 and w∈N, then, the wth moments of reverse residual life of PoiGHL distribution is given by*

M˜w(t)=1F(t)∑r=0w∑r=0∞wr(−1)rtw−rCi,j∂r∂vrB(φ(t);a(i+1),j+1),

*where F(.) is the cdf of PoiGHL, $\varphi \left(t\right)=1-e^{-\lambda t}$, v=j+1, and $C_{i,j}$ is given in Proposition 1.*

**Proof.**  M˜w(t)=E[(t−X)w|X≤t]=1F(t)∫0t(t−X)wf(x)dx=1F(t)∑r=0wwr(−1)rtw−r∫0txrf(x)dx=1F(t)∑r=0wwr(−1)rtw−rψr(t). ☐

### 2.4. Order Statistics

Order statistics and their moments play a significant role in the reliability study and quality control; it is also a useful tool in the non-parametric statistic. The density of the *j*th order statistic $X_{j:n}$, say $f_{x_{j:n}}$, in a random sample of size *n* from the PoiGHL distribution can be obtain as follows

$$\begin{array}{cc} f_{x_{j:n}}\left(x;a,\alpha ,\lambda \right) & =\frac{n!}{(j-1)!(n-j)!}f(x){\left(F\left(x\right)\right)}^{j-1}{\left(1-F\left(x\right)\right)}^{n-j},\\ & =\sum_{m=0}^{n-j}\frac{n!\;{\left(-1\right)}^{m}}{(j-1)!(n-j-m)!\;m!}f(x)F^{j+m-1}\left(x\right). \end{array}$$

but Fj+m−1(x)=∑k=0j+m−1(−1)kj+m−1k(1−e−λ)j+m−1e−λk1−e−αx1+e−αxa. Substituting $F^{j+m-1}\left(x\right)$ and f(x) above, then some algebra, we have

(19) $$f_{x_{j:n}}\left(x;a,\alpha ,\lambda \right)=\sum_{m=0}^{n-j}\sum_{k=0}^{j+m-1}\Delta_{j,k,m,n}\left(\lambda \right)f\left(x;\alpha ,\lambda \left(k+1\right),a\right),$$

where Δj,k,m,n(λ)=n!(−1)m+k(1−e−λ(k+1))j+m−1k(j−1)!(n−j−m)!m!(k+1)(1−e−λ)j+m and f(x;α,λ(k+1),a) is the density function of PoiGHL(α,λ(k+1),a), therefore, the *r*th moments of the *j*th-order statistics can be computed using Equation (16) as

(20) $$E\left[X_{j:n}\right]=\sum_{m=0}^{n-j}\sum_{k=0}^{j+m-1}\Delta_{i,j,k,m,n}^{*}\left(\lambda \right)\;C_{i,l}^{*}\;B_{0r}\left(a\left(i+1\right),j+1\right)\;\;\;\;\;\;\;\;r\in N,$$

where $C_{i,l}^{*}=\sum_{l=0}^{\infty }\frac{w_{i}{\left(-1\right)}^{r}\Gamma \left(a\left(i+1\right)+l+1\right)}{\alpha^{r+1}l!\Gamma \left(a\left(i+1\right)+1\right)}$ and Δi,j,k,m,n*(λ)=2αaλi+1(k+1)in!(−1)m+k+ij+m−1k(j−1)!(n−j−m)!m!(1−e−λ)j+mi!

### 2.5. Probability Weighted Moments

Probability-weighted moments (PWM) can be defined as expectations of functions of a random variable provided the ordinary moments of the random variable exist. It has various applications especially in parameters estimation of a distribution whose inverse form cannot be expressed explicitly and often used when maximum likelihood estimation (MLE) fails or difficult to compute, they may also be applied as starting values for MLEs. The estimation based on PWM is often considered to be superior to standard moment method of estimation. The PWM method was originally considered by [21]. The applications and details of PWM can be found in [22,23]. For a random variable *X* the probability weighted moments is defined by ${\bar{\delta }}_{r,s}=E\left[X^{r}F^{s}\left(X\right)\right]$, where F(x) and f(x) are the cdf and pdf of *X*. Now, we obtain the PWMs of the PoiGHL as follows.

$${\bar{\delta }}_{r,s}=E\left[X^{r}F^{s}\left(X\right)\right]=\int_{0}^{\infty }x^{r}F^{s}\left(x\right)f\left(x\right)dx\;\;\;\;r,s\in N,$$

we can express $F^{s}\left(x\right)$ as

Fs(x)=∑i=0s(−1)isi(1−e−λ)se−λi1−e−αx1+e−αxa

therefore,

$${\bar{\delta }}_{r,s}=b_{i}\int_{0}^{\infty }\frac{x^{r}e^{-\alpha x}{\left(1-e^{-\alpha x}\right)}^{a-1}}{{\left(1+e^{-\alpha x}\right)}^{a+1}}e^{-\lambda \left(i+1\right){\left(\frac{1-e^{-\alpha x}}{1+e^{-\alpha x}}\right)}^{a}}dx$$

where bi=∑i=0s2aαλ(−1)isi(1−e−λ)s+1. By the exponential expansion and letting $u=1-e^{-\alpha x}$ we get

$$\begin{array}{cc} {\bar{\delta }}_{r,s} & =\sum_{j=0}^{\infty }\sum_{k=0}^{\infty }\omega_{i,j,k}\int_{0}^{1}\ln^{r}\left(1-u\right)u^{a(i+1)-1}{\left(1-u\right)}^{k}du\\ & =\sum_{j=0}^{\infty }\sum_{k=0}^{\infty }\omega_{i,j,k}B_{0r}\left(a\left(i+1\right),k+1\right), \end{array}$$

where ωi,j,k=(−1)j+rbiλj(i+1)j−(a(i+1)+1)jαr+1j!.

### 2.6. Log-PoiGHL Distribution

In this subsection, we proposed the log-PoiGHL distribution (LPoiGHL) and discussed the relationship of PoiGHL with some other popular model.

The transformation of a random variable *X* from a uni-variate distribution to Y=logX has been studied for most of the classical probability models. The application of the logX transformation in modeling censored data using linear location-scale regression modeling can be found in [24] for log-beta Burr III (LBBurr III). However, there are still many extension of classical distributions for which the logX transform has not been considered. Here, we derive the expression for the density and the cumulative distribution of the log−PoiGHL.

**Figure 5:**
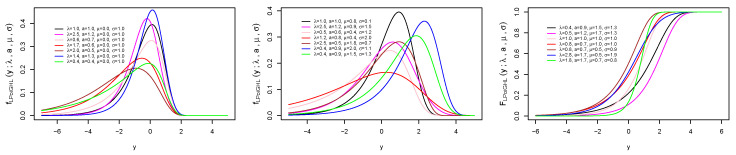
Plots of the pdf and cdf of the LPoiGHL for various parameter values: f(y;λ,a,μ,σ) (**left**) and f(y;λ,a,μ,σ) (**middle**), F(y;λ,a,μ,σ) (**right**).

Let *X* be a random variable having the PoiGHL given by (5), the random variable Y=σlogX has a log-PoiGHL distribution, whose density is parametrized by $\alpha =e^{-\frac{\mu }{\sigma }}$. The cdf and the corresponding pdf of the LPoiGHL(λ,a,μ,σ) can be presented as

(21) $$F_{LPoiGHL}\left(y\right)=\frac{1-e^{-\lambda {\left(\frac{1-e^{-e^{\frac{y-\mu }{\sigma }}}}{1+e^{-e^{\frac{y-\mu }{\sigma }}}}\right)}^{a}}}{1-e^{-\lambda }},\;\;\;\;\;\lambda ,a,\sigma >0,\;y,\mu \in R,$$

and

(22) $$f_{LPoiGHL}\left(y\right)=\frac{2\;a\lambda \;e^{\frac{y-\mu }{\sigma }}\;e^{-e^{\frac{y-\mu }{\sigma }}}\;{\left(1-e^{-e^{\frac{y-\mu }{\sigma }}}\right)}^{a-1}}{\sigma \left(1-e^{-\lambda }\right)\;{\left(1+e^{-e^{\frac{y-\mu }{\sigma }}}\right)}^{a+1}}\;e^{-\lambda {\left(\frac{1-e^{-e^{\frac{y-\mu }{\sigma }}}}{1+e^{-e^{\frac{y-\mu }{\sigma }}}}\right)}^{a}},\;\lambda ,a,\sigma >0,y,\mu \in R,$$

respectively. The parameter *a* is the shape parameter, λ scaling (weight) parameter, μ location parameter, and σ is a dispersion parameter. Hence, if X∼PoiGHL(α,λ,a) then Y=σlogX∼LPoiGHL(λ,a,μ,σ). Figure 5 provide the plots of the density and cdf of LPoiGHL(λ,a,μ,σ) for some values of parameters. Moreover, we can define the standardized random variable Z∼LPoiGHL(λ,a,μ,σ) as Z=(Y−μ)/σ, where Z∈R. The density and cdf of *Z* are given by

(23) $$f_{LPoiGHL}\left(z\right)=\frac{2\;a\lambda \;e^{z}\;e^{-e^{z}}\;{\left(1-e^{-e^{z}}\right)}^{a-1}}{\left(1-e^{-\lambda }\right)\;{\left(1+e^{-e^{z}}\right)}^{a+1}}\;e^{-\lambda {\left(\frac{1-e^{-e^{z}}}{1+e^{-e^{z}}}\right)}^{a}}$$

and

(24) $$F_{LPoiGHL}\left(z\right)=\frac{1-e^{-\lambda {\left(\frac{1-e^{-e^{z}}}{1+e^{-e^{z}}}\right)}^{a}}}{1-e^{-\lambda }}.$$

LPoiGHL can be use in the estimation of univariate survival function for censored data via linear location-scale regression modeling defined by $y_{i}=\mu_{i}+\sigma z_{i},\;i=1,\cdots ,n$ (see, [24]), where $y_{i}∼LPoiGHL$ model given by (22), $\mu_{i}=x_{i}^{T}\beta$ is the location of $y_{i}$, $z_{i}$ is the random error with density in (23), $x_{i}=\left(x_{i1},x_{i2},\cdots ,x_{ip}\right)$ is a vector of known explanatory random variables associated with $y_{i}$ and $\beta ={\left(\beta_{1},\beta_{2},\cdots ,\beta_{p}\right)}^{T}$ is a (p<n) vector of the unknown regression parameters. The location parameter vector $\mu ={\left(\mu_{i},\mu_{2},\cdots ,\mu_{n}\right)}^{T}$ is known as the model matrix of rank *p*; $\mu =X^{T}\beta$, where $X={\left(x_{1},x_{2},\cdots ,x_{n}\right)}^{T}$.

#### Some Related Distributions

We suppose that X is a random variable with the PoiGHL in (5), then some distributions that are related to the PoiGHL can be obtained as follows.

**Proposition** **5.** *Let X be a random variable with pdf in (5), if $Y={\left[\frac{1}{\sigma }\log\left(\frac{1+e^{-\alpha x}}{2e^{-\alpha x}}\right)\right]}^{\frac{1}{\beta }}$, then: (i) Y has the exponentiated Weibull Poisson (EWP) with parameters a,β,σ,λ>0, highlighted in [9] (ii) if a=1, Y is Weibull Poisson (WP) [25,26] (iii) if β=1, Y is exponentiated exponential Poisson (EEP) [27] (iv) if a=β=1, Y is exponential Poisson (EP) [1] (v) if $\sigma =(1-e^{-\theta })\gamma$, with θ,γ>0, then Y has Poisson generalized new-weibull (PGNW), where the cdf of the generalized new weibull is $G\left(x\right)={\left(1-e^{-\left(1-e^{-\theta }\right)\gamma x^{\beta }}\right)}^{a}$ (vi) if $\sigma =(1-e^{-\theta })\gamma$, with θ,γ>0, and a=1, then Y has Poisson new-weibull (PNW) (vii) if $\sigma =(1-e^{-\theta })\gamma$, with θ,γ>0, and β=1, then Y is the Poisson exponentiated Erlang-truncated exponential (PEETE) [28] (viii) if $\sigma =(1-e^{-\theta })\gamma$, with θ,γ>0, and a=β=1, then Y is the Poisson Erlang-truncated exponential (PETE) [28].*

**Proof.**  Let $Y={\left[\frac{1}{\sigma }\log\left(\frac{1+e^{-\alpha x}}{2e^{-\alpha x}}\right)\right]}^{\frac{1}{\beta }}$. Then $X=\frac{1}{\alpha }\log\left(2e^{\sigma y^{\beta }}-1\right)$, and the Jacobian of the transformation is $J=\frac{2\beta \sigma y^{\beta -1}}{\alpha (2-e^{-\sigma y^{\beta }})}$, therefore,

$$f\left(x\right)=\frac{a\beta \sigma \lambda y^{\beta -1}e^{-\sigma y^{\beta }}}{1-e^{-\lambda }}{\left(1-e^{-\sigma y^{\beta }}\right)}^{a-1}e^{-\lambda {\left(1-e^{-\sigma y^{\beta }}\right)}^{a}},\;\;y,a,\beta ,\sigma ,\lambda >0,$$

which is the pdf of the exponentiated Weibull Poisson (EWP). The proof of (ii)-(viii) follow similar. ☐

**Proposition** **6.** *Let X be a random variable with pdf in (5), If  $Y=\frac{1}{\gamma }\log\left[\left(\gamma \log\left(\frac{1+e^{-\alpha x}}{2e^{-\alpha x}}\right)+\theta \right)/\theta \right]$, then Y has the Poisson generalized Gompertz (PGG) with parameters a,θ,γ,λ>0, mention in [11] and if a=1 we have Gompertz Poisson (GP) [29].*

**Proof.**  Let $Y=\frac{1}{\gamma }\log\left[\left(\gamma \log\left(\frac{1+e^{-\alpha x}}{2e^{-\alpha x}}\right)+\theta \right)/\theta \right]$. Then $X=\frac{1}{\alpha }\log\left(2e^{\frac{\theta }{\gamma }\left(e^{\gamma y}-1\right)}-1\right)$, and the Jacobian of the transformation is $J=\frac{2\theta e^{\gamma y}}{\alpha \left(2-e^{-\frac{\theta }{\gamma }\left(e^{\gamma y}-1\right)}\right)}$ therefore,

$$f\left(x\right)=\frac{a\theta \lambda e^{\gamma y}e^{-\frac{\theta }{\gamma }\left(e^{\gamma y}-1\right)}}{1-e^{-\lambda }}{\left(1-e^{-\frac{\theta }{\gamma }\left(e^{\gamma y}-1\right)}\right)}^{a-1}e^{-\lambda {\left(1-e^{-\frac{\theta }{\gamma }\left(e^{\gamma y}-1\right)}\right)}^{a}},\;\;\;y,a,\theta ,\gamma ,\lambda >0,$$

which is the pdf of the Poisson generalized Gompertz and if a=1 is Gompertz Poisson. ☐

## 3. Entropies and Kullback-Leibler Divergence

In this section, we study the two most popular entropy measure known as the Shannon and Renyi entropies, we also examine their behavior numerically. The Kullback-Leibler (K||L) divergence of random variables with PoiGHL distribution is computed. The following lemma and proposition are used in the computations of these measures.

**Lemma** **1.** *For $\beta_{1},\beta_{2},\beta_{3}\in R$, let*

$$A\left(\beta_{1},\beta_{2},\beta_{3}\right)=\int_{0}^{\infty }\frac{e^{-\alpha x}{\left(1-e^{-\alpha x}\right)}^{\beta_{1}}}{{\left(1+e^{-\alpha x}\right)}^{\beta_{2}}}e^{-\lambda {\left(\frac{1-e^{-\alpha x}}{1+e^{-\alpha x}}\right)}^{\beta_{3}}}dx,$$

*then,*

$$A\left(\beta_{1},\beta_{2},\beta_{3}\right)=\sum_{i=0}^{\infty }\sum_{j=0}^{\infty }\;D_{i,j}\;B\left(\beta_{1}+\beta_{3}i+1,j+1\right).$$

*where, Di,j=(−1)iλi−(β2+β3i)jαi!.*

**Proof.**  Let,

$$\begin{array}{cc} A(\beta_{1},\beta_{2},\beta_{3}) & =\int_{0}^{\infty }\frac{e^{-\alpha x}{\left(1-e^{-\alpha x}\right)}^{\beta_{1}}}{{\left(1+e^{-\alpha x}\right)}^{\beta_{2}}}e^{-\lambda {\left(\frac{1-e^{-\alpha x}}{1+e^{-\alpha x}}\right)}^{\beta_{3}}}dx,\\ & =\sum_{i=0}^{\infty }\frac{{\left(-\lambda \right)}^{i}}{i!}\int_{0}^{\infty }\frac{e^{-\alpha x}{\left(1-e^{-\alpha x}\right)}^{\beta_{1}+\beta_{3}i}}{{\left(1+e^{-\alpha x}\right)}^{\beta_{2}+\beta_{3}i}}dx, \end{array}$$

let $u=1-e^{-\alpha x}$ then by applying expansion to approximate the denominator we get,

$$\begin{array}{cc} A(\beta_{1},\beta_{2},\beta_{3}) & =\sum_{i=0}^{\infty }\sum_{j=0}^{\infty }D_{i,j}\int_{0}^{1}u^{\beta_{1}+\beta_{3}i}{\left(1-u\right)}^{j}du,\\ & =\sum_{i=0}^{\infty }\sum_{j=0}^{\infty }\;D_{i,j}\;B\left(\beta_{1}+\beta_{3}i+1,j+1\right). \end{array}$$

where, Di,j=(−1)iλi−(β2+β3i)jαi!. ☐

**Proposition** **7.** *Let X be a random variable with pdf given by (5), then,*

$$\begin{array}{cc} E\left(\log(1-e^{-\alpha X})\right) & =\frac{2a\alpha \lambda }{\left(1-e^{-\lambda }\right)}\frac{\partial }{\partial t}{A\left(a+t-1,a+1,a\right)|}_{t=0},\\ E\left(\log(1+e^{-\alpha X})\right) & =\frac{2a\alpha \lambda }{\left(1-e^{-\lambda }\right)}\frac{\partial }{\partial t}{A\left(a-1,a-t+1,a\right)|}_{t=0},\\ E\left({\left(\frac{1-e^{-\alpha X}}{1+e^{-\alpha X}}\right)}^{a}\right) & =\frac{2a\alpha \lambda }{\left(1-e^{-\lambda }\right)}A\left(2a-1,2a+1,a\right). \end{array}$$

**Proof.**  By using Lemma 1 above. ☐

### 3.1. Shannon and Renyi Entropies

Entropy is the degree of disorder or randomness in a system, it has many applications in various fields of science, engineering, and finance such as statistical mechanics, thermodynamics, economics, biomedical studies among others. Entropy of a random variable *X* with density function f(x) is a measure of variation of uncertainty. Here, we consider the two important entropies known as the Shannon and Renyi entropies. The Shannon entropy measure is defined by E[−logf(X)]. The Shannon entropy of *X* with PoiGHL can be computed by considering the Lemma 1 and Proposition 7 as follows.

$$\begin{array}{cc} E\left[-\log f(X)\right] & =\log\left(\frac{1-e^{-\lambda }}{2\;a\;\alpha \;\lambda }\right)+\alpha E\left[X\right]-\left(a-1\right)E\left[\log\left(1-e^{-\alpha x}\right)\right]+\left(a+1\right)E\left[\log\left(1+e^{-\alpha x}\right)\right]\\ & +\lambda E\left[{\left(\frac{1-e^{-\alpha x}}{1+e^{-\alpha x}}\right)}^{a}\right], \end{array}$$

by Proposition 7 we have

(25) $$\begin{array}{cc} E\left[-\log f(X)\right] & =\log\left(\frac{1-e^{-\lambda }}{2\;a\;\alpha \;\lambda }\right)+\alpha \mu_{1}-\frac{2a(a-1)\alpha \lambda }{\left(1-e^{-\lambda }\right)}\frac{\partial }{\partial t}{A\left(a+t-1,a+1,a\right)|}_{t=0}\\ & +\frac{2a(a+1)\alpha \lambda }{\left(1-e^{-\lambda }\right)}\frac{\partial }{\partial t}{A\left(a-1,a-t+1,a\right)|}_{t=0}+\frac{2a\alpha \lambda^{2}}{\left(1-e^{-\lambda }\right)}A\left(2a-1,2a+1,a\right). \end{array}$$

It can be seen from Table 2 that the Shannon entropy is decreasing as α,λ,a increases.

For a random variable *X* with pdf (5), the Renyi entropy is defined by $I_{R(\rho )}={\left(1-\rho \right)}^{-1}\log\left[\int_{0}^{\infty }f^{\rho }\left(x\right)dx\right]$, where ρ>0 and ρ≠1, notice that Shannon entropy is a special case of Renyi entropy as ρ→1. We begin with

$$\begin{array}{c} \int_{0}^{\infty }f^{\rho }\left(x\right)dx=\frac{2^{\rho }a^{\rho }\alpha^{\rho }\lambda^{\rho }}{{\left(1-e^{-\lambda }\right)}^{\rho }}\sum_{i=0}^{\infty }\frac{{\left(-1\right)}^{i}\lambda^{i}\rho^{i}}{i!}\int_{0}^{\infty }e^{-\alpha \rho x}\frac{{\left(1-e^{-\alpha x}\right)}^{\rho (a-1)+ai}}{{\left(1+e^{-\alpha x}\right)}^{2\rho +\rho (a-1)+ai}}dx, \end{array}$$

by letting $u=1-e^{-\alpha x}$ and expansion of the denominator we have

$$\int_{0}^{\infty }f^{\rho }\left(x\right)dx=\frac{2^{\rho }a^{\rho }\alpha^{\rho -1}}{{\left(1-e^{-\lambda }\right)}^{\rho }}\sum_{i=0}^{\infty }\sum_{j=0}^{\infty }\;\tau_{i,j}\;\int_{0}^{1}u^{\rho (a-1)+ai}{\left(1-u\right)}^{\rho +j-1}du,$$

where $\tau_{i,j}=\frac{{\left(-1\right)}^{i}\lambda^{i+\rho }\rho^{i}\Gamma \left(a\left(i+\rho \right)+\rho +j\right)}{i!\;j!\;\Gamma (a(i+\rho )+\rho )}$, therefore,

$$\int_{0}^{\infty }f^{\rho }\left(x\right)dx=\frac{2^{\rho }a^{\rho }\alpha^{\rho -1}}{{\left(1-e^{-\lambda }\right)}^{\rho }}\sum_{i=0}^{\infty }\sum_{j=0}^{\infty }\;\tau_{i,j}\;B\left(a\left(i+\rho \right)-\rho +1,\rho +j\right).$$

Thus, the Renyi entropy is

$$\begin{array}{cc} I_{R(\rho )} & =\rho {\left(1-\rho \right)}^{-1}\log\left[\frac{2a}{1-e^{-\lambda }}\right]-\log\alpha +\frac{1}{\left(1-\rho \right)}\log\left[\sum_{i=0}^{\infty }\sum_{j=0}^{\infty }\;\tau_{i,j}\;B\left(a\left(\rho +i\right)-\rho +1,\rho +j+1\right)\right]. \end{array}$$

Table 3 show that if ρ increases for a fixed value of α,λ,a the entropy decreases, while if α,λ,a and ρ are increasing the entropy is decreasing.

### 3.2. Kullback-Leibler Divergence

Now, we compute the Kullback-Leibler (K||L) divergence for the PoiGHL distributions. The K||L-divergence is a fundamental equation of information theory that measures the proximity of two probability distributions. It is also called the information divergence and relative entropy. For convenience, we choose the scale parameter α to be common. For a random variables $X_{1}∼PoiGHL\left(\alpha ,\lambda_{1},a_{1}\right)$ and $X_{2}∼PoiGHL\left(\alpha ,\lambda_{2},a_{2}\right)$, the $K||L=\int_{0}^{\infty }f_{1}\left(x\right)\log\frac{f_{1}\left(x\right)}{f_{2}\left(x\right)}dx$.

$$K||L=\int_{0}^{\infty }f_{1}\left(x\right)\log f_{1}\left(x\right)dx-\int_{0}^{\infty }f_{1}\left(x\right)\log f_{2}\left(x\right)dx,$$

but $\int_{0}^{\infty }f_{1}\left(x\right)\log f_{1}\left(x\right)dx=E_{f_{1}}\left[\log f_{1}\left(x\right)\right]$ and $\int_{0}^{\infty }f_{1}\left(x\right)\log f_{2}\left(x\right)dx=E_{f_{1}}\left[\log f_{2}\left(x\right)\right]$, therefore, the integrals can be computed by considering (25) and applying Proposition 7:

$$\begin{array}{cc} E_{f_{1}}\left[\log f_{1}\left(X\right)\right] & =\log\left(\frac{2\;a_{1}\;\alpha \;\lambda_{1}}{1-e^{-\lambda_{1}}}\right)-\alpha \mu_{1}+\frac{2a_{1}\left(a_{1}-1\right)\alpha \lambda_{1}}{\left(1-e^{-\lambda_{1}}\right)}\frac{\partial }{\partial t}A\left(a_{1}+t-1,a_{1}+1,a_{1}\right){|}_{t=0}\\ & -\frac{2a_{1}\left(a_{1}+1\right)\alpha \lambda_{1}}{\left(1-e^{-\lambda_{1}}\right)}\frac{\partial }{\partial t}A\left(a_{1}-1,a_{1}-t+1,a_{1}\right){|}_{t=0}-\frac{2a_{1}\alpha \lambda_{1}^{2}}{\left(1-e^{-\lambda_{1}}\right)}A\left(2a_{1}-1,2a_{1}+1,a_{1}\right), \end{array}$$

and

$$\begin{array}{cc} E_{f_{1}}\left[\log f_{2}\left(X\right)\right] & =\log\left(\frac{2\;a_{2}\;\alpha \;\lambda_{2}}{1-e^{-\lambda_{2}}}\right)-\alpha \mu_{1}+\frac{2a_{1}\left(a_{2}-1\right)\alpha \lambda_{1}}{\left(1-e^{-\lambda_{1}}\right)}\frac{\partial }{\partial t}A\left(a_{1}+t-1,a_{1}+1,a_{1}\right){|}_{t=0}\\ & -\frac{2a_{1}\left(a_{2}+1\right)\alpha \lambda_{1}}{\left(1-e^{-\lambda_{1}}\right)}\frac{\partial }{\partial t}A\left(a_{1}-1,a_{1}-t+1,a_{1}\right){|}_{t=0}\\ & -\frac{2a_{1}\alpha \lambda_{1}\lambda_{2}}{\left(1-e^{-\lambda_{1}}\right)}A\left(a_{1}+a_{2}-1,a_{1}+a_{2}+1,a_{1}\right), \end{array}$$

therefore,

$$\begin{array}{cc} K||L & =\int_{0}^{\infty }f_{1}\left(x\right)\log f_{1}\left(x\right)dx-\int_{0}^{\infty }f_{1}\left(x\right)\log f_{2}\left(x\right)dx,\\ & =\log\left(\frac{a_{1}\;\lambda_{1}\left(1-e^{-\lambda_{2}}\right)}{a_{2}\;\lambda_{2}\left(1-e^{-\lambda_{1}}\right)}\right)+\frac{2a_{1}\left(a_{1}-a_{2}\right)\alpha \lambda_{1}}{\left(1-e^{-\lambda_{1}}\right)}\frac{\partial }{\partial t}A\left(a_{1}+t-1,a_{1}+1,a_{1}\right){|}_{t=0}\\ & +\frac{2a_{1}\left(a_{2}-a_{1}\right)\alpha \lambda_{1}}{\left(1-e^{-\lambda_{1}}\right)}\frac{\partial }{\partial t}A\left(a_{1}-1,a_{1}-t+1,a_{1}\right){|}_{t=0}-\frac{2a_{1}\alpha \lambda_{1}^{2}}{\left(1-e^{-\lambda_{1}}\right)}A\left(2a_{1}-1,2a_{1}+1,a_{1}\right)\\ & +\frac{2a_{1}\alpha \lambda_{1}\lambda_{2}}{\left(1-e^{-\lambda_{1}}\right)}A\left(a_{1}+a_{2}-1,a_{1}+a_{2}+1,a_{1}\right). \end{array}$$

## 4. Characterization of PoiGHL Sub Model by Truncated Moments

Characterizations of probability distributions based on certain statistics are very essential in statistical inference and stochastic modeling. Ref. [30] discussed the characterizations via mean residual life and failure rates functions of absolutely continuous random variables. Ref. [31] characterized distributions by truncated moments. Ref. [32] investigate characterization of distributions by the moments of residual life. Recently, the characterization of Lindley distribution based on conditional expectations was discussed by [33]. Here we are able to characterize the sub model of PoiGHL distribution (i.e if a=1) known as the half logistic Poisson (HLP) [12] based on some certain functional conditional expectations. The probability density, cumulative distribution function of the HLP with α,λ,x>0 are

(26) $$\begin{array}{cc} f(x) & =\frac{2\alpha \;\lambda \;e^{-\alpha x}}{\left(1-e^{-\lambda }\right)\;{\left(1+e^{-\alpha x}\right)}^{2}}e^{-\lambda \left(\frac{1-e^{-\alpha x}}{1+e^{-\alpha x}}\right)}, \end{array}$$

(27) $$\begin{array}{cc} F(x) & =\frac{1-e^{-\lambda \left(\frac{1-e^{-\alpha x}}{1+e^{-\alpha x}}\right)}}{1-e^{-\lambda }}, \end{array}$$

respectively. Now, we provide supportive lemma for the characterization based on left truncated moments.

**Lemma** **2.** *Suppose that the random variable X has an absolutely continuous c.d.f F(x) with F(0)=0, F(x)>0∀x>0, with density function $f\left(x\right)=F^{\prime }\left(x\right)$ and failure rate h(x)=f(x)/[1−F(x)]. Let Q(x) be a continuous function in x>0 and E[Q(X)]<∞. If E[Q(X)|X≥x]=Ψ(x)h(x),x>0, where Ψ(x) is a differentiable function in x>0, then*

$$f\left(x\right)=\Lambda \exp\left[-\int_{0}^{x}\frac{Q\left(y\right)+\Psi^{\prime }\left(y\right)}{\Psi (y)}dy\right],\;\;x>0,$$

*where Λ>0 is a normalizing constant.*

**Proof.**  Since,

$$E\left[Q\left(X\right)|X\geq x\right]=\frac{1}{1-F(x)}\int_{x}^{\infty }Q\left(y\right)f\left(y\right)dy,$$

it follow that,

$$\int_{x}^{\infty }Q\left(y\right)f\left(y\right)dy=\Psi \left(x\right)f\left(x\right),$$

differentiating both side we get

$$-Q\left(x\right)f\left(x\right)=\Psi \left(x\right)f^{\prime }\left(x\right)+\Psi^{\prime }\left(x\right)f\left(x\right),$$

this implies

(28) $$f^{\prime }\left(x\right)+\left(\frac{Q\left(x\right)+\Psi^{\prime }\left(x\right)}{\Psi (x)}\right)f\left(x\right)=0$$

which is first order homogeneous linear differential equation w.r.t f(x). From the general solution of (28) we have

$$f\left(x\right)=\Lambda \exp\left[-\int_{0}^{x}\frac{Q\left(y\right)+\Psi^{\prime }\left(y\right)}{\Psi (y)}dy\right],\;\;x>0,$$

where Λ is normalizing constant. ☐

Next, we characterized HLP based on Lemma 2.

**Theorem** **2.** *Suppose that the random variable X has an absolutely continuous c.d.f F(x) with F(0)=0, F(x)>0∀x>0, with density function $f\left(x\right)=F^{\prime }\left(x\right)$ and failure rate h(x)=f(x)/[1−F(x)]. Assume that $E\left[e^{-\lambda \left(\frac{1-e^{-\alpha X}}{1+e^{-\alpha X}}\right)}\right]<\infty$ for all α,λ,x>0, then X∼HLP if and only if*

$$E\left[e^{-\lambda \left(\frac{1-e^{-\alpha X}}{1+e^{-\alpha X}}\right)}|X\geq x\right]=\frac{𝓱\left(x\right)\;\left[e^{-2\lambda \left(\frac{1-e^{-\alpha x}}{1+e^{-\alpha x}}\right)}-e^{-2\lambda }\right]}{4\alpha \;\lambda \;\frac{e^{-\alpha x}}{{\left(1+e^{-\alpha x}\right)}^{2}}\;e^{-\lambda \left(\frac{1-e^{-\alpha x}}{1+e^{-\alpha x}}\right)}},\;\;\;x>0.$$

**Proof.**  See Appendix A.1. ☐

The following lemma described the characterization of a distribution by right truncated moment.

**Lemma** **3.** *Suppose that the random variable X has an absolutely continuous c.d.f F(x) with F(0)=0, F(x)>0∀x>0, with density function $f\left(x\right)=F^{\prime }\left(x\right)$ and reverse failure rate r(x)=f(x)/F(x). Let Q(x) be a continuous function in x>0 and E[Q(X)]<∞. If E[Q(X)|X≤x]=V(x)r(x),x>0, where V(x) is a differentiable function in x>0, then*

$$f\left(x\right)=\Lambda \exp\left[-\int_{0}^{x}\frac{V^{\prime }\left(y\right)-Q\left(y\right)}{V(y)}dy\right],\;\;x>0,$$

*where Λ>0 is a normalizing constant.*

**Proof.**  Since,

$$E\left[Q\left(X\right)|X\leq x\right]=\frac{1}{F(x)}\int_{0}^{x}Q\left(y\right)f\left(y\right)dy,$$

it follow that,

$$\int_{0}^{x}Q\left(y\right)f\left(y\right)dy=V\left(x\right)f\left(x\right),$$

differentiating both side we get

$$Q\left(x\right)f\left(x\right)=V\left(x\right)f^{\prime }\left(x\right)+V^{\prime }\left(x\right)f\left(x\right),$$

this implies,

(29) $$f^{\prime }\left(x\right)+\left(\frac{V^{\prime }\left(x\right)-Q\left(x\right)}{V(x)}\right)f\left(x\right)=0,$$

which is first order homogeneous linear differential equation w.r.t f(x). From the general solution of (29) we have

$$f\left(x\right)=\Lambda \exp\left[-\int_{0}^{x}\frac{V^{\prime }\left(y\right)-Q\left(y\right)}{V(y)}dy\right],\;\;x>0,$$

where Λ is normalizing constant.

☐

Now, we provide the characterization of HLP based on Lemma 3.

**Theorem** **3.** *Suppose that the random variable X has an absolutely continuous c.d.f F(x) with F(0)=0, F(x)>0∀x>0, with density function $f\left(x\right)=F^{\prime }\left(x\right)$ and reverse failure rate r(x)=f(x)/F(x). Assume that $E\left[e^{-\lambda \left(\frac{1-e^{-\alpha X}}{1+e^{-\alpha X}}\right)}\right]<\infty$ for all α,λ,x>0, then X∼HLP if and only if*

$$E\left[e^{-\lambda \left(\frac{1-e^{-\alpha X}}{1+e^{-\alpha X}}\right)}|X\leq x\right]=\frac{r\left(x\right)\left[1-e^{-2\lambda \left(\frac{1-e^{-\alpha x}}{1+e^{-\alpha x}}\right)}\right]e^{\lambda \left(\frac{1-e^{-\alpha x}}{1+e^{-\alpha x}}\right)}}{4\alpha \;\lambda \;\frac{e^{-\alpha x}}{{\left(1+e^{-\alpha x}\right)}^{2}}},\;\;\;x>0.$$

**Proof.**  See Appendix A.2. ☐

## 5. Estimation and Inference

Estimation of the unknown parameters of the PoiGHL distribution by the method of maximum likelihood is established in this section. Let $x_{1},x_{2},x_{3},\cdots ,x_{n}$ be a random sample of size n obtained from the PoiGHL distribution. Let $\theta ={\left(\alpha ,\lambda ,a\right)}^{T}$ be a vector of parameters, the maximum likelihood estimates (MLEs) of θ, say $\hat{\theta }={\left(\hat{\alpha },\hat{\lambda },\hat{a}\right)}^{T}$ are obtained via the maximization of the log-likelihood function (logℓ(θ)) given by

(30) $$\begin{array}{cc} \log\ell (\theta ) & =n\log2+n\log a+n\log\alpha +n\log\lambda -n\log\left(1-e^{-\lambda }\right)-\alpha \sum_{i=1}^{n}x_{i}\\ & +\left(a-1\right)\sum_{i=1}^{n}\log\left(1-e^{-\alpha x_{i}}\right)-\left(a+1\right)\sum_{i=1}^{n}\log\left(1+e^{-\alpha x_{i}}\right)-\lambda \sum_{i=1}^{n}{\left(\frac{1-e^{-\alpha x_{i}}}{1+e^{-\alpha x_{i}}}\right)}^{a}. \end{array}$$

To find the MLEs $\hat{\theta }={\left(\hat{a},\hat{\alpha },\hat{\lambda }\right)}^{T}$, we need to obtain the solutions of the partial derivative of logℓ(θ) with respect to α,λ, and *a* i.e $\frac{\partial \ell }{\partial a}=\frac{\partial \ell }{\partial \alpha }=\frac{\partial \ell }{\partial \lambda }=0$, this can only be achieved by numerical technique such as Newton–Raphson technique using mathematical packages such as R and Mathematica etc. The partial derivative of logℓ(θ) are

(31) $$\begin{array}{cc} \frac{\partial \ell }{\partial \alpha } & =\frac{n}{\alpha }-\sum_{i=1}^{n}x_{i}+\left(a-1\right)\sum_{i=1}^{n}\frac{x_{i}e^{-\alpha x_{i}}}{1-e^{-\alpha x_{i}}}+\left(a+1\right)\sum_{i=1}^{n}\frac{x_{i}e^{-\alpha x_{i}}}{1+e^{-\alpha x_{i}}}-2a\lambda \sum_{i=1}^{n}\frac{x_{i}e^{-\alpha x_{i}}\;{\left(1-e^{-\alpha x_{i}}\right)}^{a-1}}{{\left(1+e^{-\alpha x_{i}}\right)}^{a+1}} \end{array}$$

(32) $$\begin{array}{cc} \frac{\partial \ell }{\partial \lambda } & =\frac{n}{\lambda }-\frac{ne^{-\lambda }}{\left(1-e^{-\lambda }\right)}-\sum_{i=1}^{n}{\left(\frac{1-e^{-\alpha x_{i}}}{1+e^{-\alpha x_{i}}}\right)}^{a} \end{array}$$

(33) $$\begin{array}{cc} \frac{\partial \ell }{\partial a} & =\frac{n}{a}+\sum_{i=1}^{n}\log\left(1-e^{-\alpha x_{i}}\right)-\sum_{i=1}^{n}\log\left(1+e^{-\alpha x_{i}}\right)-\lambda \sum_{i=1}^{n}{\left(\frac{1-e^{-\alpha x_{i}}}{1+e^{-\alpha x_{i}}}\right)}^{a}\log\left(\frac{1-e^{-\alpha x_{i}}}{1+e^{-\alpha x_{i}}}\right) \end{array}$$

For the interval estimation and hypothesis tests of the parameters we required K(θ) the 3×3 Fisher information matrix which is given by $K\left(\theta \right)=-E\left(\partial^{2}\left(\log\ell (\theta )\right)/\partial \theta \partial \theta^{T}\right)$. The approximate of the MLEs of θ, the $\hat{\theta }$, can be approximated as $N_{3}\left(0,K{\left(\hat{\theta }\right)}^{-1}\right)$ under the usual condition for the parameters in the interior of the parameter space but not on the boundary. The approximate asymptotic distribution of $\sqrt{n}\left(\hat{\alpha }-\alpha ,\hat{\lambda }-\lambda ,\hat{a}-a\right)$ is three dimensional normal distribution with zero means and covariance matrix $K^{-1}\left(\theta \right)$. The asymptotic behavior is also valid as $K\left(\theta \right)=\lim_{n\to \infty }n^{-1}J_{n}\left(\theta \right)$, where $J_{n}\left(\theta \right)$ is a unit information matrix evaluated at $\hat{\theta }={\left(\hat{a},\hat{\alpha },\hat{\lambda }\right)}^{T}$. The asymptotic confidence interval for each parameter θ can be determine using 100(1−ϵ)% confidence interval as $ACI_{\alpha }=\left(\hat{\alpha }-\omega_{\frac{\epsilon }{2}}\Xi_{11},\hat{\alpha }+\omega_{\frac{\epsilon }{2}}\Xi_{11}\right),$ $ACI_{\lambda }=\left(\hat{\lambda }-\omega_{\frac{\epsilon }{2}}\Xi_{22},\hat{\lambda }+\omega_{\frac{\epsilon }{2}}\Xi_{22}\right),$ and $ACI_{a}=\left(\hat{a}-\omega_{\frac{\epsilon }{2}}\Xi_{33},\hat{a}+\omega_{\frac{\epsilon }{2}}\Xi_{33}\right),$ where $\Xi_{rr}$ is the square root of the diagonal elements of $J_{n}{\left(\theta \right)}^{-1}$, for r=1,2,3 and $\omega_{\frac{\epsilon }{2}}$ is the quantile $\left(1-\frac{\epsilon }{2}\right)$ of the standard normal distribution. The elements of J(θ) are in Appendix B.

$$J\left(\theta \right)=-\left[\begin{array}{ccc} J_{\alpha \alpha } & J_{\alpha \lambda } & J_{\alpha a}\\ J_{\alpha \lambda } & J_{\lambda \lambda } & J_{a\lambda }\\ J_{\alpha a} & J_{a\lambda } & J_{aa} \end{array}\right]$$

Next, we can compared PoiGHL with its sub model by conducting a likelihood ratio test (LR). Let consider $\hat{\theta }$ and $\overset{ˇ}{\theta }$ be the unrestricted and restricted MLEs of θ respectively, then the LR test between the null hypothesis $H_{0}:\theta_{1}=\theta_{1}^{0}$ versus alternative hypothesis $H_{1}:\theta_{1}\neq \theta_{1}^{0}$ is the $w=-2(\ell \left(\overset{ˇ}{\theta }\right)-\ell \left(\hat{\theta }\right))$, where $\overset{ˇ}{\theta }$ is under $H_{0}$ and $\hat{\theta }$ under the complete distribution i.e PoiGHL. The LR test under $H_{0}$ is asymptotically distributed as $\chi_{l}^{2}$ with degree of freedom *l*, where *l* is the difference in parameter dimension between the unrestricted model and the restricted model. The LR test rejects $H_{0}$ at level ξ whenever $w>\chi_{l,1-\xi }^{2}$, where $\chi_{l,1-\xi }^{2}$ is the 1−ξ quantile of Chi-square distribution with degree of freedom *l*.

Now, we study the existence and uniqueness of the MLEs as discussed in [29,34,35] among others.

**Proposition** **8.** *Let $j_{1}\left(\alpha ;\lambda ,a,x\right)$ be the right hand side of (31), given that λ and a are true values of the parameters, then $j_{1}\left(\alpha ;\lambda ,a,x\right)=0$ has at least one root for a≥1.*

**Proof.**  Let $j_{1}\left(\alpha ;\lambda ,a,x\right)$ be the right hand of (31), then $\lim_{\alpha \to 0}j_{1}=\infty$ and $\lim_{\alpha \to \infty }j_{1}=-\sum_{i=1}^{n}x_{i}$, thus, $j_{1}$ is a decreasing from non-negative or zero to negative, hence, $j_{1}=0$ has at least one root. ☐

**Proposition** **9.** *Let $j_{2}\left(\lambda ;\alpha ,a,x\right)$ be the right hand side of (32), given that α and a are true values of the parameters, then $j_{2}\left(\lambda ;\alpha ,a,x\right)=0$ has at least one root if $n^{-1}\sum_{i=1}^{n}{\left(\frac{1-e^{-\alpha x_{i}}}{1+e^{-\alpha x_{i}}}\right)}^{a}<\frac{1}{2}$.*

**Proof.**  Let $j_{2}\left(\lambda ;\alpha ,a,x\right)$ be the right hand of (32), and let $m_{2}\left(\lambda \right)=\frac{1}{\lambda }-\frac{e^{-\lambda }}{1-e^{-\lambda }}$. $\lim_{\lambda \to \infty }m_{2}\left(\lambda \right)=0$, therefore, $\lim_{\lambda \to \infty }j_{2}=-\sum_{i=1}^{n}{\left(\frac{1-e^{-\alpha x_{i}}}{1+e^{-\alpha x_{i}}}\right)}^{a}<0$, also, $\lim_{\lambda \to 0}m_{2}\left(\lambda \right)=\frac{1}{2}$, therefore, $\lim_{\lambda \to 0}j_{2}=\frac{n}{2}-\sum_{i=1}^{n}{\left(\frac{1-e^{-\alpha x_{i}}}{1+e^{-\alpha x_{i}}}\right)}^{a}$, where $\lim_{\lambda \to 0}j_{2}>0$ only if $n^{-1}\sum_{i=1}^{n}{\left(\frac{1-e^{-\alpha x_{i}}}{1+e^{-\alpha x_{i}}}\right)}^{a}<\frac{1}{2}$.

Thus, for $n^{-1}\sum_{i=1}^{n}{\left(\frac{1-e^{-\alpha x_{i}}}{1+e^{-\alpha x_{i}}}\right)}^{a}<\frac{1}{2}$, $j_{2}$ decreases from non-negative or zero to negative, hence $j_{2}=0$ has at least one root. ☐

**Proposition** **10.** *Let $j_{3}\left(a;\alpha ,\lambda ,x\right)$ be the right hand side of (33), given that α and λ<1 are true values of the parameters, then $j_{3}\left(a;\alpha ,\lambda ,x\right)=0$ has a unique root in*

*$\left(\frac{n}{\sum_{i=1}^{n}\log\left(\frac{1+e^{-\alpha x_{i}}}{1-e^{-\alpha x_{i}}}\right)},\frac{n}{\left(1-\lambda \right)\sum_{i=1}^{n}\log\left(\frac{1+e^{-\alpha x_{i}}}{1-e^{-\alpha x_{i}}}\right)}\right)$.*

**Proof.**  Let $j_{3}\left(a;\alpha ,\lambda ,x\right)$ be the right hand of (33), let $m_{3}\left(a\right)=-\lambda \sum_{i=1}^{n}{\left(\frac{1-e^{-\alpha x_{i}}}{1+e^{-\alpha x_{i}}}\right)}^{a}\log\left(\frac{1-e^{-\alpha x_{i}}}{1+e^{-\alpha x_{i}}}\right)$, then $\lim_{a\to 0}m_{3}=-\lambda \sum_{i=1}^{n}\log\left(\frac{1-e^{-\alpha x_{i}}}{1+e^{-\alpha x_{i}}}\right)$, therefore, $j_{3}<\frac{n}{a}+\sum_{i=1}^{n}\log\left(\frac{1-e^{-\alpha x_{i}}}{1+e^{-\alpha x_{i}}}\right)+\lim_{a\to 0}m_{3}=\frac{n}{a}+\left(1-\lambda \right)\sum_{i=1}^{n}\log\left(\frac{1-e^{-\alpha x_{i}}}{1+e^{-\alpha x_{i}}}\right)$, thus, $j_{3}<0$ only if $a>\frac{n}{\left(1-\lambda \right)\sum_{i=1}^{n}\log\left(\frac{1+e^{-\alpha x_{i}}}{1-e^{-\alpha x_{i}}}\right)}$. on the other hand, $\lim_{a\to \infty }m_{3}=0$, therefore, $j_{3}>\frac{n}{a}+\sum_{i=1}^{n}\log\left(\frac{1-e^{-\alpha x_{i}}}{1+e^{-\alpha x_{i}}}\right)+\lim_{a\to \infty }m_{3}=\frac{n}{a}+\sum_{i=1}^{n}\log\left(\frac{1-e^{-\alpha x_{i}}}{1+e^{-\alpha x_{i}}}\right)$, thus, $j_{3}>0$ only if $a<\frac{n}{\sum_{i=1}^{n}\log\left(\frac{1+e^{-\alpha x_{i}}}{1-e^{-\alpha x_{i}}}\right)}$, hence $j_{3}=0$ has a root in the interval

$\left(\frac{n}{\sum_{i=1}^{n}\log\left(\frac{1+e^{-\alpha x_{i}}}{1-e^{-\alpha x_{i}}}\right)},\frac{n}{\left(1-\lambda \right)\sum_{i=1}^{n}\log\left(\frac{1+e^{-\alpha x_{i}}}{1-e^{-\alpha x_{i}}}\right)}\right)$. This is analogous to [1,3]. To show the uniqueness, we show that $j_{3}^{\prime }<0$ and it follow from $J_{aa}$ in the element of information matrix in Appendix B. ☐

### 5.1. Simulation Study

Simulation results are obtained to assess the performance of the proposed maximum likelihood method. We generate 10,000 samples of size n=(30,50,100,150,200,300), the estimated values, standard deviations (sd), bias and mean square error (MSE) of the estimators are computed using R-software. The results presented in Table 4 indicated that the performance of the MLE is quite good, it is clear that the estimated values of the parameters converge to their actual values as the sample size increases. The standard deviations and the mean square error decrease as the sample size increases, it is also noted that the bias is negative in some cases.

## 6. Stress-Strength Reliabilty Analysis

Let the random variables $X_{1}$ and $X_{2}$ be independent that follow PoiGHL$\left(\alpha ,\lambda_{1},a_{1}\right)$ and PoiGHL$\left(\alpha ,\lambda_{2},a_{2}\right)$ respectively. In reliability analysis, the stress-strength model describes the life of a component which has a random strength $X_{1}$ that is subjected to a random stress $X_{2}$. If $X_{1}>X_{2}$ the component will function satisfactorily and when $X_{2}>X_{1}$ the component will fail because the stress applied exceed the strength. The reliability of a component $R=P\left(X_{1}>X_{2}\right)=\int_{0}^{\infty }f_{1}\left(x;\alpha ,\lambda_{1},a_{1}\right)F_{2}\left(x;\alpha ,\lambda_{2},a_{2}\right)dx$, has many applications in different fields of engineering such as maintenance in electric power, and in study of fatigue failure of a components or structures etc.

The reliability *R* when $X_{1}$ and $X_{2}$ are independent random variables with the same univariate distributions, and its algebraic formula has been analyzed for most of the popular (or classical) distributions. For example, the estimation of $P[X_{1}<X_{2}]$ for generalized Pareto distribution has been considered by [36] and three-parameter generalized exponential distribution [37] among others. Estimation of $P[X_{1}<X_{2}]$ from logistic random variable [38], and Laplace distribution [39]. However, there are still many other models especially the extensions of the classical distributions for which the form of *R* has not been derived. Now, we obtain the expression of R for PoiGHL and analyze it the special case when α=1 and common λ for convenience. We start by

$$\begin{array}{cc} \int_{0}^{\infty }f_{1}\left(x\right)F_{2}\left(x\right)dx & =\frac{1}{\left(1-e^{-\lambda_{2}}\right)}-(\frac{2a_{1}\alpha \lambda_{1}}{\left(1-e^{-\lambda_{1}}\right)\left(1-e^{-\lambda_{2}}\right)}\\ & \times \int_{0}^{\infty }\frac{e^{-\alpha x}e^{-\lambda_{1}{\left(\frac{1-e^{-\alpha x}}{1+e^{-\alpha x}}\right)}^{a_{1}}}}{{\left(1+e^{-\alpha x}\right)}^{2}}{\left(\frac{1-e^{-\alpha x}}{1+e^{-\alpha x}}\right)}^{a_{1}-1}e^{-\lambda_{2}{\left(\frac{1-e^{-\alpha x}}{1+e^{-\alpha x}}\right)}^{a_{2}}}dx), \end{array}$$

by expansion of $e^{-\lambda_{1}{\left(\frac{1-e^{-\alpha x}}{1+e^{-\alpha x}}\right)}^{a_{1}}}$ and $e^{-\lambda_{2}{\left(\frac{1-e^{-\alpha x}}{1+e^{-\alpha x}}\right)}^{a_{2}}}$, then letting $u=1-e^{-\alpha x}$, we get

$$\int_{0}^{\infty }f_{1}\left(x\right)F_{2}\left(x\right)dx=\frac{1}{\left(1-e^{-\lambda_{2}}\right)}-{\hat{d}}_{i,j}\int_{0}^{1}\frac{u^{a_{1}\left(i+1\right)+a_{2}j-1}}{{\left(1+\left(1-u\right)\right)}^{a_{1}\left(i+1\right)+a_{2}j+1}}du$$

where ${\hat{d}}_{i,j}=\frac{2a_{1}}{\left(1-e^{-\lambda_{1}}\right)\left(1-e^{-\lambda_{2}}\right)}\sum_{i=0}^{\infty }\sum_{j=0}^{\infty }\frac{{\left(-1\right)}^{i+j}\lambda_{1}^{i+1}\lambda_{2}^{j}}{i!j!}$. Using the expansion of ${\left(1+\left(1-u\right)\right)}^{-(a_{1}\left(i+1\right)+a_{2}j+1)}$ we have

$$\int_{0}^{\infty }f_{1}\left(x\right)F_{2}\left(x\right)dx=\frac{1}{\left(1-e^{-\lambda_{2}}\right)}-\sum_{k=0}^{\infty }{\hat{d}}_{i,j,k}^{*}\int_{0}^{1}u^{a_{1}\left(i+1\right)+a_{2}j-1}{\left(1-u\right)}^{k}du,$$

where ${\hat{d}}_{i,j,k}^{*}=\frac{{\hat{d}}_{i,j}\Gamma \left(a_{1}\left(i+1\right)+a_{2}j+k+1\right)}{k!\Gamma (a_{1}\left(i+1\right)+a_{2}j+1)}$, hence,

$$R=\frac{1}{\left(1-e^{-\lambda_{2}}\right)}-\sum_{k=0}^{\infty }{\hat{d}}_{i,j,k}^{*}B\left(a_{1}\left(i+1\right)+a_{2}j,k+1\right).$$

Notice that *R* is independent of α.

### 6.1. Estimation of R with a Common Parameter λ

In this subsection, we compute R with common parameter λ. For convenience, we choose to consider two parameter PoiGHL that is when the scale parameter α=1. The MLEs of R and the asymptotic confidence interval of R are discussed

**Proposition** **11.** *Let $X_{1}∼PoiGHL\left(a_{1},\lambda \right)$ and $X_{2}∼PoiGHL\left(a_{2},\lambda \right)$ be independent random variables, then the reliability $R=P(X_{2}<X_{1})$ is given as*

(34) $$R=\frac{1}{\left(1-e^{-\lambda }\right)}-\sum_{i,j,k=0}^{\infty }M_{i,j,k}B\left(a_{1}\left(i+1\right)+a_{2}j,k+1\right)$$

*where $M_{i,j,k}=\frac{2a_{1}\lambda {\left(-\lambda \right)}^{i+j}\Gamma \left(a_{1}\left(i+1\right)+a_{2}j+k+1\right)}{{\left(1-e^{-\lambda }\right)}^{2}i!j!k!\Gamma \left(a_{1}\left(i+1\right)+a_{2}j+1\right)}$.*

#### 6.1.1. MLE and Asymptotic Confidence Interval of R

Let $X_{1},\;X_{2},\;X_{3},\cdots ,X_{n}$ be an independent random sample of size *n* from the PoiGHL($a_{1},\lambda$) population, and let $Y_{1},\;Y_{2},\;Y_{3},\cdots ,Y_{m}$ be an independent random sample of size *m* from the PoiGHL($a_{2},\lambda$) population. We wish to estimate the parameters $a_{1},\;a_{2}$, and λ by method of maximum likelihood estimation. The log-likelihood function (logℓ(Θ)) of the observed samples is given by

(35) $$\begin{array}{cc} \log\ell (\Theta )= & \left(n+m\right)\log2+n\log a_{1}+m\log a_{2}+\left(n+m\right)\log\lambda -\left(n+m\right)\log\left(1-e^{-\lambda }\right)\\ & +\left(a_{1}-1\right)\sum_{i=1}^{n}\log\left(1-e^{-x}\right)-\left(a_{1}+1\right)\sum_{i=1}^{n}\log\left(1+e^{-x}\right)-\lambda \sum_{i=1}^{n}{\left(\frac{1-e^{-x}}{1+e^{-x}}\right)}^{a_{1}}\\ & +\left(a_{2}-1\right)\sum_{j=1}^{m}\log\left(1-e^{-y}\right)-\left(a_{2}+1\right)\sum_{j=1}^{m}\log\left(1+e^{-x}\right)-\lambda \sum_{j=1}^{m}{\left(\frac{1-e^{-x}}{1+e^{-x}}\right)}^{a_{2}} \end{array}$$

The MLEs of $\Theta ={\left(a_{1},a_{2},\lambda \right)}^{T}$ say, $\hat{\Theta }={\left(\hat{a_{1}},\hat{a_{2}},\hat{\lambda }\right)}^{T}$ can be obtain numerically by the solution of the nonlinear system (36) to (38) obtained by from (35).

(36) $$\begin{array}{cc} \frac{\partial \ell }{\partial a_{1}} & =\frac{n}{a_{1}}+\sum_{i=1}^{n}\log\left(1-e^{-x}\right)-\sum_{i=1}^{n}\log\left(1+e^{-x}\right)-\lambda \sum_{i=1}^{n}{\left(\frac{1-e^{-x}}{1+e^{-x}}\right)}^{a_{1}}\log\left(\frac{1-e^{-x}}{1+e^{-x}}\right) \end{array}$$

(37) $$\begin{array}{cc} \frac{\partial \ell }{\partial a_{2}} & =\frac{m}{a_{2}}+\sum_{j=1}^{m}\log\left(1-e^{-y}\right)-\sum_{j=1}^{m}\log\left(1+e^{-y}\right)-\lambda \sum_{j=1}^{m}{\left(\frac{1-e^{-x}}{1+e^{-x}}\right)}^{a_{2}}\log\left(\frac{1-e^{-x}}{1+e^{-x}}\right) \end{array}$$

(38) $$\begin{array}{cc} \frac{\partial \ell }{\partial \lambda } & =\frac{n+m}{\lambda }-\frac{\left(n+m\right)e^{-\lambda }}{1-e^{-\lambda }}-\sum_{i=1}^{n}{\left(\frac{1-e^{-x}}{1+e^{-x}}\right)}^{a_{1}}-\sum_{j=1}^{m}{\left(\frac{1-e^{-x}}{1+e^{-x}}\right)}^{a_{2}} \end{array}$$

The existence and the uniqueness of the MLEs can be analyzed as follows

**Proposition** **12.** *Let $q_{1}\left(a_{1};\lambda ,x\right)$ be the right hand side of (36), given that λ<1 is a true values of the parameters, then $q_{1}\left(a_{1};\lambda ,x\right)=0$ has a unique root in $\left(\frac{n}{\sum_{i=1}^{n}\log\left(\frac{1+e^{-x_{i}}}{1-e^{-x_{i}}}\right)},\frac{n}{\left(1-\lambda \right)\sum_{i=1}^{n}\log\left(\frac{1+e^{-x_{i}}}{1-e^{-x_{i}}}\right)}\right)$.*

**Proof.**  Follow similar to Proposition 10. ☐

**Proposition** **13.** *Let $q_{2}\left(a_{2};\lambda ,y\right)$ be the right hand side of (37), given that λ<1 is a true values of the parameters, then $q_{2}\left(a_{2};\lambda ,y\right)=0$ has a unique root in $\left(\frac{m}{\sum_{j=1}^{m}\log\left(\frac{1+e^{-y_{j}}}{1-e^{-y_{j}}}\right)},\frac{m}{\left(1-\lambda \right)\sum_{j=1}^{m}\log\left(\frac{1+e^{-y_{j}}}{1-e^{-y_{j}}}\right)}\right)$.*

**Proof.**  Follow similar to Proposition 10. ☐

**Proposition** **14.** *Let $q_{3}\left(\lambda ;a_{1},a_{2},x,y\right)$ be the right hand side of (38), given that $a_{1}$ and $a_{2}$ are true values of the parameters, then $q_{3}\left(\lambda ;a_{1},a_{2},x,y\right)=0$ has at least one root if ${\left(n+m\right)}^{-1}\left(\sum_{i=1}^{n}{\left(\frac{1-e^{-x_{i}}}{1+e^{-x_{i}}}\right)}^{a_{1}}+\sum_{j=1}^{m}{\left(\frac{1-e^{-y_{j}}}{1+e^{-y_{j}}}\right)}^{a_{2}}\right)<\frac{1}{2}$.*

**Proof.**  Let $q_{3}\left(\lambda ;a_{1},a_{2},x,y\right)$ be the right hand of (38), and let $w\left(\lambda \right)=\frac{1}{\lambda }-\frac{e^{-\lambda }}{1-e^{-\lambda }}$. $\lim_{\lambda \to \infty }w\left(\lambda \right)=0$, therefore, $\lim_{\lambda \to \infty }q_{3}=-\left(\sum_{i=1}^{n}{\left(\frac{1-e^{-x_{i}}}{1+e^{-x_{i}}}\right)}^{a_{1}}+\sum_{j=1}^{m}{\left(\frac{1-e^{-y_{j}}}{1+e^{-y_{j}}}\right)}^{a_{2}}\right)<0$, also, $\lim_{\lambda \to 0}w\left(\lambda \right)=\frac{1}{2}$, therefore, $\lim_{\lambda \to 0}q_{3}=\frac{n+m}{2}-\left(\sum_{i=1}^{n}{\left(\frac{1-e^{-x_{i}}}{1+e^{-x_{i}}}\right)}^{a_{1}}+\sum_{j=1}^{m}{\left(\frac{1-e^{-y_{j}}}{1+e^{-y_{j}}}\right)}^{a_{2}}\right)$, where $\lim_{\lambda \to 0}q_{3}>0$ only if ${\left(n+m\right)}^{-1}\left(\sum_{i=1}^{n}{\left(\frac{1-e^{-x_{i}}}{1+e^{-x_{i}}}\right)}^{a_{1}}+\sum_{j=1}^{m}{\left(\frac{1-e^{-y_{j}}}{1+e^{-y_{j}}}\right)}^{a_{2}}\right)<\frac{1}{2}$.

Thus, for ${\left(n+m\right)}^{-1}\left(\sum_{i=1}^{n}{\left(\frac{1-e^{-x_{i}}}{1+e^{-x_{i}}}\right)}^{a_{1}}+\sum_{j=1}^{m}{\left(\frac{1-e^{-y_{j}}}{1+e^{-y_{j}}}\right)}^{a_{2}}\right)<\frac{1}{2}$, $q_{3}$ decreases from non-negative or zero to negative, hence $q_{3}=0$ has at least one root. ☐

Next, the asymptotic distribution of the $\hat{\Theta }={\left(\hat{a_{1}},\;\hat{a_{2}},\lambda \right)}^{T}$ and asymptotic confidence interval of $\hat{R}$ are established. We denote the expected Fisher information matrix by $I\left(\Theta \right)=-E\left(\frac{\partial^{2}\left(\log\ell (\Theta )\right)}{\partial \Theta \partial \Theta^{T}}\right)$. The elements $\left(\frac{\partial^{2}\left(\log\ell \right)}{\partial \Theta \partial \Theta^{T}}\right)$ are given in Appendix C.1.

$$I\left(\Theta \right)=-\left[\begin{array}{ccc} u_{11} & u_{12} & u_{13}\\ u_{21} & u_{22} & u_{23}\\ u_{31} & u_{32} & u_{33} \end{array}\right]$$

where, some elements $u_{i,j},i,j=1,2,3$ can be obtain by considering Lemma 1 at α=1.

$$\begin{array}{cc} u_{11} & =-\frac{n}{a_{1}^{2}}-\frac{2a_{1}\lambda^{2}n}{\left(1-e^{-\lambda }\right)}\frac{\partial^{2}}{\partial t^{2}}A^{*}\left(2a_{1}+t-1,2a_{1}+t+1,a_{1}\right){\;|}_{t=0},\\ u_{22} & =-\frac{2a_{2}\lambda m}{\left(1-e^{-\lambda }\right)}\frac{\partial }{\partial t}A^{*}\left(2a_{2}+t-1,2a_{2}+t+1,a_{2}\right){\;|}_{t=0},\\ u_{33} & =-\frac{\left(n+m\right)}{\lambda^{2}}+\frac{\left(n+m\right)e^{-\lambda }}{{\left(1-e^{-\lambda }\right)}^{2}},\\ u_{13} & =u_{31}=-\frac{2a_{1}\lambda n}{\left(1-e^{-\lambda }\right)}\frac{\partial }{\partial t}A^{*}\left(2a_{1}+t-1,2a_{1}+t+1,a_{1}\right){\;|}_{t=0},\\ u_{23} & =u_{32}=-\frac{2a_{2}\lambda m}{\left(1-e^{-\lambda }\right)}\frac{\partial }{\partial t}A^{*}\left(2a_{2}+t-1,2a_{2}+t+1,a_{2}\right){\;|}_{t=0},\\ u_{12} & =u_{21}=0, \end{array}$$

where the computation of $A^{*}$ is similar to *A* in Lemma 1 at α=1. The above computation are given in Appendix C.2. The asymptotic variances and covariances of the estimators $\hat{a_{1}}$, $\hat{a_{2}}$ and $\hat{\lambda }$ is needed to compute the variance of the estimator of $\hat{R}$. The variance covariance matrix is the $I^{-1}$ as

$$I^{-1}=\left[\begin{array}{ccc} Var\left(\hat{a_{1}}\right) & Cov(\hat{a_{1}},\hat{a_{2}}) & Cov(\hat{a_{1}},\hat{\lambda })\\ Cov(\hat{a_{2}},\hat{a_{1}}) & Var\left(\hat{a_{2}}\right) & Cov(\hat{a_{2}},\hat{\lambda })\\ Cov(\hat{\lambda },\hat{a_{1}}) & Cov(\hat{\lambda },\hat{a_{2}}) & Var\left(\hat{\lambda }\right) \end{array}\right]$$

**Theorem** **4.** *As n→∞, m→∞ and n/m→p, then, $\left[\sqrt{n}\left(\hat{a_{1}}-a_{1}\right),\sqrt{m}\left(\hat{a_{2}}-a_{2}\right),\sqrt{n}\left(\hat{\lambda }-\lambda \right)\right]\to N_{3}\left(0,B^{-1}\left(\Theta \right)\right)$, where*

$$B\left(\Theta \right)=\left[\begin{array}{ccc} \lim_{m,n\to \infty }\frac{u_{11}}{n} & 0 & \lim_{m,n\to \infty }\frac{u_{13}}{n}\\ 0 & \lim_{m,n\to \infty }\frac{u_{22}}{m} & \lim_{m,n\to \infty }\frac{\sqrt{p}\;u_{23}}{n}\\ \lim_{m,n\to \infty }\frac{u_{31}}{n} & \lim_{m,n\to \infty }\frac{\sqrt{p}\;u_{32}}{n} & \lim_{m,n\to \infty }\frac{u_{33}}{n} \end{array}\right]$$

**Proof.**  The proof follows from the asymptotic normality of maximum likelihood estimation. ☐

We intended to construct the confidence interval of $\hat{R}$, which requires to determine the variance of $\hat{R}$. The asymptotic variance of $\hat{R}$ is defined by

(39) $$\begin{array}{cc} Var(\hat{R}) & ={\left(\frac{\partial R}{\partial a_{1}}\right)}^{2}Var\left(a_{1}\right)+{\left(\frac{\partial R}{\partial a_{2}}\right)}^{2}Var\left(a_{2}\right)+{\left(\frac{\partial R}{\partial \lambda }\right)}^{2}Var\left(\lambda \right)\\ & +2\left(\frac{\partial R}{\partial a_{1}}\frac{\partial R}{\partial a_{2}}\right)Cov\left(a_{1},a_{2}\right)+2\left(\frac{\partial R}{\partial a_{1}}\frac{\partial R}{\partial \lambda }\right)Cov\left(a_{1},\lambda \right)\\ & +2\left(\frac{\partial R}{\partial a_{2}}\frac{\partial R}{\partial \lambda }\right)Cov\left(a_{2},\lambda \right) \end{array}$$

let us derive the expressions for the $\frac{\partial R}{\partial a_{1}},\frac{\partial R}{\partial a_{2}}$ and $\frac{\partial R}{\partial \lambda }$, the numerical values of these derivatives can be computed using mathematical packages, we use R-software. In similar way, we consider Lemma 1 and the computations are given in Appendix C.3.

$$\begin{array}{cc} \frac{\partial R}{\partial a_{2}} & =\frac{2a_{1}\lambda^{2}}{{\left(1-e^{-\lambda }\right)}^{2}}\sum_{k=0}^{\infty }\frac{{\left(-\lambda \right)}^{k}}{k!}\frac{\partial }{\partial t}A^{*}\left(a_{1}\left(k+1\right)+a_{2}+t-1,a_{1}\left(k+1\right)+a_{2}+t+1,a_{2}\right){|}_{t=0},\\ \frac{\partial R}{\partial a_{1}} & =-\frac{2\lambda }{{\left(1-e^{-\lambda }\right)}^{2}}\sum_{k=0}^{\infty }\frac{{\left(-\lambda \right)}^{k}}{k!}A^{*}\left(a_{1}+a_{2}k-1,a_{1}+a_{2}k+1,a_{1}\right)\\ & -\frac{2a_{1}\lambda }{{\left(1-e^{-\lambda }\right)}^{2}}\sum_{k=0}^{\infty }\frac{{\left(-\lambda \right)}^{k}}{k!}\frac{\partial }{\partial t}A^{*}\left(a_{1}+a_{2}k+t-1,a_{1}+a_{2}k+t+1,a_{1}\right){|}_{t=0}\\ & +\frac{2a_{1}\lambda^{2}}{{\left(1-e^{-\lambda }\right)}^{2}}\sum_{k=0}^{\infty }\frac{\partial }{\partial t}A^{*}\left(2a_{1}+a_{2}k+t-1,2a_{1}+a_{2}k+t+1,a_{1}\right){|}_{t=0}\\ \frac{\partial R}{\partial \lambda } & =-\frac{e^{-\lambda }}{{\left(1-e^{-\lambda }\right)}^{2}}-\frac{2a_{1}}{{\left(1-e^{-\lambda }\right)}^{2}}\sum_{k=0}^{\infty }\frac{{\left(-\lambda \right)}^{k}}{k!}A^{*}\left(a_{1}+a_{2}k-1,a_{1}+a_{2}k-1,a_{1}\right)\\ & +\frac{4a_{1}\lambda e^{-\lambda }}{{\left(1-e^{-\lambda }\right)}^{3}}\sum_{k=0}^{\infty }\frac{{\left(-\lambda \right)}^{k}}{k!}A^{*}\left(a_{1}+a_{2}k-1,a_{1}+a_{2}k-1,a_{1}\right)\\ & +\frac{2a_{1}\lambda }{{\left(1-e^{-\lambda }\right)}^{2}}\sum_{k=0}^{\infty }\frac{{\left(-\lambda \right)}^{k}}{k!}A^{*}\left(2a_{1}+a_{2}k-1,2a_{1}+a_{2}k-1,a_{1}\right)\\ & +\frac{2a_{1}\lambda }{{\left(1-e^{-\lambda }\right)}^{2}}\sum_{k=0}^{\infty }\frac{{\left(-\lambda \right)}^{k}}{k!}A^{*}\left(a_{1}+a_{2}\left(k+1\right)-1,a_{1}+a_{2}\left(k+1\right)-1,a_{1}\right) \end{array}$$

Once the estimated $Var(\hat{R})$ is obtained using $\hat{\Theta }$, then we can get the 95% asymptotic confidence interval of R defined by $\hat{R}\pm 1.96\sqrt{\hat{Var}\left(\hat{R}\right)}$.

#### 6.1.2. Simulation Results

We generate N=10,000 samples from $X∼PoiGHL(a_{1},\lambda )$ and $Y∼PoiGHL(a_{2},\lambda )$. The combination sample (n,m) are (20, 20), (30, 20), (30, 40) and (50, 50). The estimates of $a_{1},a_{2}$ and λ are obtained from the samples to compute $\hat{R}$. The validity of the estimation of *R* is discussed by analyzing (i) the average bias of the simulated *N* estimates of R, $Bias\left(\hat{R}\right)=\frac{1}{N}\sum_{i=1}^{N}\left({\hat{R}}_{i}-R\right)$ (ii) the average mean square error of the simulated N estimates of R, $MSE\left(\hat{R}\right)=\frac{1}{N}\sum_{i=1}^{N}{\left({\hat{R}}_{i}-R\right)}^{2}$ (iii) The average length of the asymptotic 95% confidence intervals of *R*, $ALCI=\frac{1}{N}\sum_{i=1}^{N}2\left(1.96\right)\sqrt{\hat{Var}\left(\hat{R}\right)}$.

It is clear from the Table 5, the MLE method perform consistently, as the sample sizes increases by *n* or *m* or both, it is observe that the MSE decrease for $a_{1}<a_{2}$ or $a_{1}>a_{2}$. The performance of confidence interval based on the MLEs is quite good and the ALCI decreases as the sample sizes increases. The variance $Var(\hat{R})$ is also decreasing as the sample sizes increases.

## 7. Real Data Applications

In this section, applications of PoiGHL is provided to demonstrate the usefulness of the new model in the various field of studies. The performance of PoiGHL in terms of fit is presented and its application in the stress-strength analysis is provided for illustration.

### 7.1. Application I

Here, we illustrate the superiority of the PoiGHL as compared to some other existing distributions using three real data sets. For each data set, we estimate the model’s parameters by maximum likelihood estimation, and compare the fitted models by the Akaike Information Criterion (AIC), Bayesian Information Criterion (BIC), Consistent Akaike Information Criterion (CAIC). Moreover, the goodness of fit statistics known as the Anderson-Darling (AD), Cramer-von Mises (CvM), and Kolmogorov Smirnov (KS) are considered. The AD and CvM for each model are computed using the algorithm provided in the R-package called nortest [40], while the KS is obtained by the algorithm in the R-package called GLDEX [41]. The model with the smallest value of these measures represent the data better than the other models. Further, the LR test between PoiGHL and HLP is discussed for each data. The competing distributions include: the generalized half logistic (GHL) in (1), half logistic Poisson (HLP) in (27) and

- Mc-Donald half-logistic (McHL) [42] with cdf defined by,
  $F\left(x\right)=\frac{1}{B(a,b)}\int_{0}^{{\left(\frac{1-e^{-\alpha x}}{1+e^{-\alpha x}}\right)}^{c}}w^{a-1}{\left(1-w\right)}^{b-1}dw,\;x,\alpha ,a,b,c>0$.
- Beta half-logistic (BHL) [43], $F\left(x\right)=\frac{1}{B(a,b)}\int_{0}^{\left(\frac{1-e^{-\alpha x}}{1+e^{-\alpha x}}\right)}w^{a-1}{\left(1-w\right)}^{b-1}dw,\;x,\alpha ,a,b>0.$
- Kumaraswamy half-logistic (KwHL) [44], $F\left(x\right)=1-{\left(1-{\left(\frac{1-e^{-\alpha x}}{1+e^{-\alpha x}}\right)}^{a}\right)}^{b},\;x,\alpha ,a,b>0$
- Type I half-logistic Burr X (TIHLBx) [45], $F\left(x\right)=\frac{1-{\left[1-{\left(1-e^{-{\left(\alpha x\right)}^{2}}\right)}^{\theta }\right]}^{\lambda }}{1+{\left[1-{\left(1-e^{-{\left(\alpha x\right)}^{2}}\right)}^{\theta }\right]}^{\lambda }},\;x,\alpha ,\theta ,\lambda >0.$
- Poisson odd generalize exponential-half logistic (POGE-HL) [11],
$F\left(x\right)=\frac{1-e^{-\lambda {\left(1-e^{-\alpha (\frac{e^{x}-1}{2})}\right)}^{\beta }}}{1-e^{-\lambda }},\;x,\alpha ,\beta ,\lambda >0.$
- Generalized half logistic poisson (GHLP) [46], $F\left(x\right)={\left(\frac{1-e^{-\lambda \left(\frac{1-e^{-\alpha x}}{1+e^{-\alpha x}}\right)}}{1-e^{-\lambda }}\right)}^{a},\;x,a,\alpha ,\lambda >0.$
- Power half logistic (PwHL) [15], $F\left(x\right)=1-\frac{2}{1+e^{\alpha x^{\beta }}},\;x,\alpha ,\beta >0$.
- Olapade half logistic (OHL) [16], $F\left(x\right)=1-\frac{2^{\beta }}{{\left(1+e^{\alpha x}\right)}^{\beta }},\;\;x,\alpha ,\beta >0$.
- Poisson half logistic (PHL) [13], $F\left(x\right)=\frac{e^{\lambda \left(\frac{1-e^{-\alpha x}}{1+e^{-\alpha x}}\right)}-1}{e^{\lambda }-1},\;x,\alpha ,\lambda >0$.
- Exponentiated generalized standardized half logistic (EGSHL) [47], $F\left(x\right)=\frac{{\left[1+e^{-x}\right]}^{a}-{\left[2^{a}e^{-ax}\right]}^{b}}{{\left(1+e^{-x}\right)}^{ab}},\;\;x,a,b>0.$
- Half logistic (HL) $F\left(x\right)=\frac{1-e^{-\alpha x}}{1+e^{-\alpha x}},x,\alpha >0.$

The first data set is the remission times (in months) of a random sample of 128 bladder cancer patients provided by [48] also analyzed by [46]. The data are: 0.08, 2.09, 3.48, 4.87, 6.94, 8.66, 13.11, 23.63, 0.20, 2.23, 3.52, 4.98, 6.97, 9.02, 13.29, 0.40, 2.26, 3.57, 5.06, 7.09, 9.22, 13.80, 25.74, 0.50, 2.46, 3.64, 5.09, 7.26, 9.47, 14.24, 25.82, 0.51, 2.54, 3.70, 5.17, 7.28, 9.74, 14.76, 26.31, 0.81, 2.62, 3.82, 5.32, 7.32, 10.06, 14.77, 32.15, 2.64, 3.88, 5.32, 7.39, 10.34, 14.83, 34.26, 0.90, 2.69, 4.18, 5.34, 7.59, 10.66, 15.96, 36.66, 1.05, 2.69, 4.23, 5.41, 7.62, 10.75, 16.62, 43.01, 1.19, 2.75, 4.26, 5.41, 7.63, 17.12, 46.12, 1.26, 2.83, 4.33, 5.49, 7.66, 11.25, 17.14, 79.05, 1.35, 2.87, 5.62, 7.87, 11.64, 17.36, 1.40, 3.02, 4.34, 5.71, 7.93, 11.79, 18.10, 1.46, 4.40, 5.85, 8.26, 11.98, 19.13, 1.76, 3.25, 4.50, 6.25, 8.37, 12.02, 2.02, 3.31, 4.51, 6.54, 8.53, 12.03, 20.28, 2.02, 3.36, 6.76, 12.07, 21.73, 2.07, 3.36, 6.93, 8.65, 12.63, 22.69.

The second data set from [49] also studied by [2] it consist of the failure times of 20 mechanical components. The data are: 0.067, 0.068, 0.076, 0.081, 0.084, 0.085, 0.085, 0.086, 0.089, 0.098, 0.098, 0.114, 0.114, 0.115, 0.121, 0.125, 0.131, 0.149, 0.160, 0.485.

The third data set is from [50] also studied by [51], it is the intervals in days between successive failures of a piece of 34 software with values: 9, 12, 11, 4, 7, 2, 5, 8, 5, 7, 1, 6, 1, 9, 4, 1, 3, 3, 6, 1, 11, 33, 7, 91, 2, 1, 87, 47, 12, 9, 135, 258, 16, 35.

**Figure 6:**
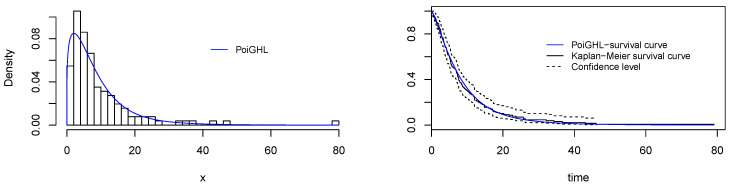
Plots of histogram with fitted PoiGHL and and estimated survival function for the first data set.

**Figure 7:**
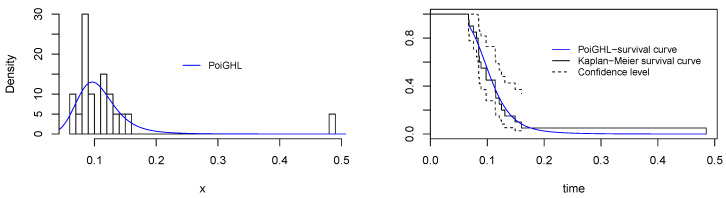
Plots of histogram with fitted PoiGHL and and estimated survival function for the second data set.

**Figure 8:**
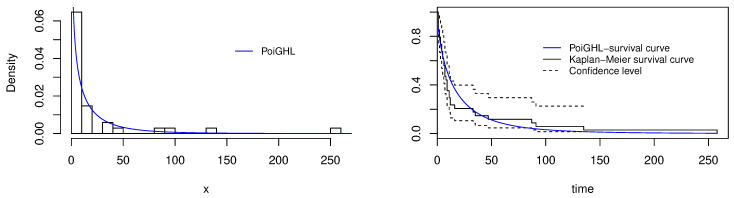
Plots of histogram with fitted PoiGHL and estimated survival function for the third data set.

**Figure 9:**
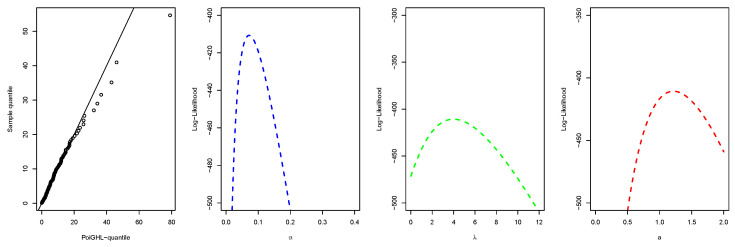
Quantile-quantile plot and plots of the profile log-likelihood for the first data set.

**Figure 10:**
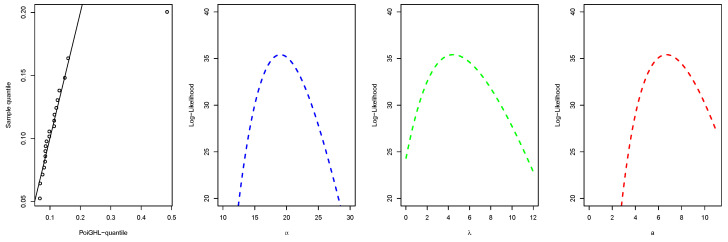
Quantile-quantile plot and plots of the profile log-likelihood for the second data set.

**Figure 11:**
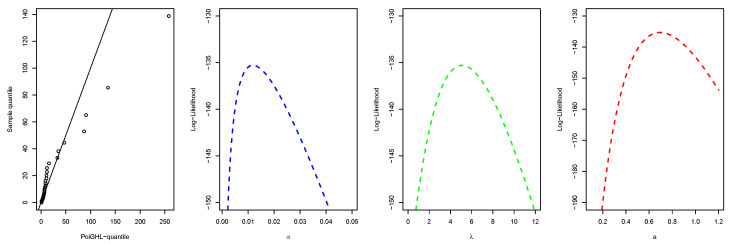
Quantile-quantile plot and plots of the profile log-likelihood for the third data set.

The MLEs and the numerical values of these measures for the PoiGHL and other competing distributions for the first, second and third data are provided in Table 6, Table 7 and Table 8 respectively. From each table it is clearly seen that PoiGHL has the smallest value of AIC, BIC, CAIC, AD, CvM and KS, thus PoiGHL represents the data set better than the other competing models. The LR test between the PoiGHL and HLP is given in Table 9 for which in all the three data set the LR test is in favor of PoiGHL. The histogram with the fitted PoiGHL density and Kaplan-Meier curve with fitted PoiGHL survival function for the first, second and third data are illustrated by Figure 6, Figure 7 and Figure 8, respectively. The quantile-quantile plot of PoiGHL and the plots of the profile log-likelihood of each parameter for each of the three data set are given in Figure 9, Figure 10 and Figure 11.

### 7.2. Application II

This subsection, demonstrated the importance of PoiGHL in stress-strength analysis. We estimate *R* and its asymptotic confidence interval by using the two data set provided by [52], say, *X* and *Y*, it is the failure stresses (in GPa) of single carbon fibers of lengths 20 mm and 50 mm, respectively. The data sets are also considered [53]:

X: 1.312, 1.314, 1.479, 1.552, 1.700, 1.803, 1.861, 1.865, 1.944, 1.958, 1.966, 1.997, 2.006, 2.021, 2.027, 2.055, 2.063, 2.098, 2.14, 2.179, 2.224, 2.240, 2.253, 2.270, 2.272, 2.274, 2.301, 2.301, 2.359, 2.382, 2.382, 2.426, 2.434, 2.435, 2.478, 2.490, 2.511, 2.514, 2.535, 2.554, 2.566, 2.57, 2.586, 2.629, 2.633, 2.642, 2.648, 2.684, 2.697, 2.726, 2.770, 2.773, 2.800, 2.809, 2.818, 2.821, 2.848, 2.88, 2.954, 3.012, 3.067, 3.084, 3.090, 3.096, 3.128, 3.233, 3.433, 3.585, 3.585 and

Y: 1.339, 1.434, 1.549, 1.574, 1.589, 1.613, 1.746, 1.753, 1.764, 1.807, 1.812, 1.84, 1.852, 1.852, 1.862, 1.864, 1.931, 1.952, 1.974, 2.019, 2.051, 2.055, 2.058, 2.088, 2.125, 2.162, 2.171, 2.172, 2.18, 2.194, 2.211, 2.27, 2.272, 2.28, 2.299, 2.308, 2.335, 2.349, 2.356, 2.386, 2.39, 2.41, 2.43, 2.431, 2.458, 2.471, 2.497, 2.514, 2.558, 2.577, 2.593, 2.601, 2.604, 2.62, 2.633, 2.67, 2.682, 2.699, 2.705, 2.735, 2.785, 3.02, 3.042, 3.116, 3.174

**Figure 12:**
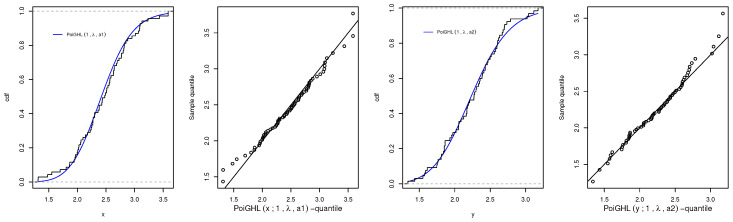
Plots of empirical with estimated PoiGHL cdf and the quantile-quantile plot for *X* and *Y*.

**Figure 13:**
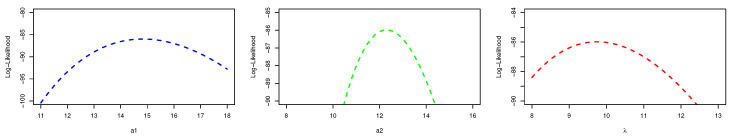
Plots of the profile log-likelihood of $a_{1},a_{2}$ and λ for the stress-strength data.

Here, we used the *R* provided in Proposition 11. Let $X∼PoiGHL(a_{1},\lambda )$ with sample size n=69 and $Y∼PoiGHL(a_{2},\lambda )$ with sample size m=65, the unknown parameters to be estimated are $a_{1},a_{2}$ and λ. The Kolmogorov-Smirnov (K-S) test is used to show how good the PoiGHL fitted the two data set. The numerical values of the MLEs, log-likelihood, and K-S are $a_{1}=14.8275,\;a_{2}=12.3005,\;\lambda =9.7478$, ℓ=−85.9813, $KS_{X}\left(p-value\right)=0.06058\left(0.9485\right)$ and $KS_{Y}\left(p-value\right)=0.0624\left(0.9482\right)$. We give a supportive plots in Figure 12 which show the plot of empirical with estimated PoiGHL cdf of *X*, the quantile-quantile plot for *X*, the plot of empirical with estimated PoiGHL cdf of *Y*, and the quantile-quantile plot for *Y*. Figure 13 is the profile log-likelihood of $a_{1},a_{2}$ and λ, which indicated that the maximum is unique.

Based on the estimation, R=0.6172, and the 95% asymptotic confidence interval of *R* is (0.4918,0.7426) with confidence length 0.2508 and the variance covariance matrix of the estimators is given below. Based on the estimators the asymptotic confidence interval is quite good indicating that PoiGHL is a good choice in reliability analysis.

$$I^{-1}=\left[\begin{array}{ccc} 1.3135027 & 0.5696736 & 1.237531\\ 0.5696736 & 0.9304918 & 1.026626\\ 1.2375312 & 1.0266256 & 2.230192 \end{array}\right]$$

## 8. Conclusions

In this work, we proposed a new three-parameter model called Poisson generalized half logistic distribution (PoiGHL). The model includes the half logistic Poisson (HLP) as a sub-model and generalized half logistic (GHL) as limiting distribution. We derived and investigated some important mathematical and statistical properties of the PoiGHL such as the closed-form expressions of *r*th moment, moment generating function, quantile function, mean deviations, Bonferroni and Lorenz curves, order statistics, moments of residual life, probability weighted moments, Shannon and Renyi entropies, and Kullback-Leibler divergence. The characterization of HLP based truncated moments is discussed. The log transform of PoiGHL and Its relationship with some known distributions is provided. Estimation of the model parameters was established based on the maximum likelihood method and examine by simulation studies. The information matrix is derived. The stress-strength analysis of random variables with PoiGHL was discussed in detail based on maximum likelihood estimation and the asymptotic variance-covariance matrix is obtained and simulation studies are used to analyze the behaviors of the estimators. We demonstrate the usefulness and superiority of PoiGHL in terms of fit and potentiality in stress-strength parameter estimation by the use of real data applications. Three real data set are used for illustration in which PoiGLH outperform some other popular distributions in terms of fit as measured by the AIC, BIC, CAIC, AD, CvM, and KS. In the estimation of the stress-strength parameter, the model performs satisfactorily in an application to real data sets as examine by MSEs and the average length of the confidence interval, indicating that PoiGHL model can be considered as a good candidate in reliability analysis.

## Figures and Tables

**Table 1:** The numerical value of the first six moments, variance, coefficient of variation, skewness and kurtosis of PoiGHL for some values of α,λ, and *a*.

α	λ	*a*	Med(X)	$\mu_{1}$	$\mu_{2}$	$\mu_{3}$	$\mu_{4}$	$\mu_{5}$	$\mu_{6}$	$\sigma^{2}$	CV	$\gamma^{3}$	$\gamma^{4}$	${\mathrm{md}}_{1}\left(X\right)$	${\mathrm{md}}_{2}\left(X\right)$
0.1	0.1	0.1	0.0152	2.1791	38.9293	1143.69	45579.83	2278781	136790766	34.18098	2.6830	4.5532	31.3715	3.2156	2.1778
0.3	0.3	0.4	0.9775	2.2510	14.7363	148.925	2005.103	33625.3	674865.9	9.6695	1.3815	2.4020	11.0717	2.2255	1.9835
0.4	0.5	0.5	0.9719	1.8872	9.3310	70.7262	713.707	8971.80	135005.1	5.7694	1.2728	2.2616	10.2491	1.7407	1.5831
0.5	0.7	0.6	0.9375	1.6356	6.4782	39.1585	315.337	3166.367	38084.38	3.8031	1.1924	2.1738	9.7942	1.4224	1.3104
0.9	0.8	0.8	0.7386	1.0948	2.4910	8.4379	37.8400	211.216	1411.52	1.2923	1.0383	1.9611	8.6776	0.8419	0.7928
1.0	1.2	1.3	0.9794	1.2504	2.6615	8.1081	32.4804	162.124	970.999	1.0979	0.8380	1.7685	7.9311	0.7810	0.7496
1.5	1.4	1.6	0.7455	0.9136	1.3203	2.6749	7.0962	23.4803	93.4016	0.4856	0.7628	1.7179	7.8155	0.5191	0.5007
1.9	1.8	2.0	0.6476	0.7682	0.8729	1.3640	2.7913	7.1688	22.2753	0.2828	0.6923	1.7226	8.0760	0.3938	0.3812
2.9	2.8	3.5	0.5397	0.5996	0.4573	0.4440	0.5505	0.8637	1.6734	0.0978	0.5215	1.7199	8.8174	0.2290	0.2235
4.0	5.0	4.5	0.3818	0.4056	0.1923	0.1080	0.0743	0.0653	0.0752	0.0278	0.4111	1.6112	9.9278	0.1229	0.1213
4.8	5.9	5.5	0.343	0.3582	0.1441	0.0657	0.0348	0.0225	0.0188	0.0158	0.3509	1.3886	9.1031	0.0938	0.0930
8.0	9.0	15	0.3078	0.3114	0.1006	0.0337	0.0117	0.0043	0.0016	0.0036	0.1919	0.6331	5.2352	0.0463	0.0462
10	19	25	0.2716	0.2718	0.0751	0.0211	0.0060	0.0017	0.0005	0.0010	0.1299	0.0577	3.1648	0.0280	0.0280

**Table 2:** The numerical value of the Shannon entropy of PoiGHL for some parameter values.

α	λ	*a*	ShannonEntropy	α	λ	*a*	ShannonEntropy	α	λ	*a*	ShannonEntropy
0.01	0.01	0.05	−10.7912	1.7	1.8	1.75	1.0116	4.1	4.3	4.2	2.0155
0.1	0.1	0.1	−3.9721	1.8	1.9	1.8	1.0458	4.3	4.5	4.3	2.1516
0.2	0.2	0.2	−0.3430	2.0	2.0	2.0	1.0856	4.5	4.8	4.9	2.1980
0.5	0.6	0.4	0.6704	2.5	2.5	2.5	1.2474	5.0	6.0	7.0	2.2810
0.6	0.7	0.5	0.8521	2.7	2.8	2.9	1.2786	7.0	8.0	8.5	3.7662
0.7	0.9	0.8	1.0452	3.1	2.9	3.0	1.5198	8.0	9.5	10	4.4776
0.9	0.9	0.9	1.0032	3.4	3.1	3.4	1.6384	18	13	15	13.2729
1.0	1.0	1.0	0.9930	3.6	3.5	3.6	1.7517	19	15	18	14.0397
1.2	1.3	1.5	0.9005	3.9	3.8	3.9	1.9155	20	25	21	14.8347
1.5	1.6	1.7	0.9375	4.0	4.2	4.1	1.9585	30	35	40	23.7911

**Table 3:** The numerical value of the Renyi entropy of PoiGHL for some values ρ and (α,λ,a).

ρ	(0.1,0.1,0.9)	(0.4,0.3,0.95)	(0.5,0.5,1.0)	(1.1,1.0,2.0)	(1.5,1.7,2.5)	(2.0,2.5,3.0)	(2.7,3.1,4.0)	(4.0,5.0,7)	(10,20,25)	(20,30,35)
0.1	4.9083	3.5180	3.2903	2.5368	2.2091	1.8922	1.5776	1.1016	−0.6298	−1.6882
0.4	3.9700	2.5734	2.3386	1.6470	1.7067	1.3694	1.0423	0.5108	−1.3035	−2.2009
0.5	3.8503	2.4527	2.2168	1.5383	1.1741	0.8047	0.4574	-0.1326	−1.7326	−2.5517
0.7	3.6868	2.2881	2.0506	1.3928	1.0197	0.6407	0.2880	-0.3072	−1.8409	−2.6551
0.9	3.5773	2.1781	1.9401	1.2977	0.9186	0.5340	0.1014	-0.4155	−1.9135	−2.7256
1.1	3.4971	2.0980	1.8597	1.2296	0.8461	0.4581	-0.0018	-0.4901	−1.9668	−2.7778
1.5	3.3844	1.9864	1.7487	1.1367	0.7475	0.3559	-0.0822	-0.5882	−2.0417	−2.8514
2.0	3.2906	1.8950	1.6589	1.0626	0.6694	0.2758	-0.2476	-0.6641	−2.1033	−2.9122
4.5	3.0647	1.6889	1.4643	0.9059	0.5057	0.1101	-0.3001	-0.8210	−2.2397	−3.0474
15	2.6944	1.4645	1.3387	0.7740	0.3701	-0.0258	-0.3831	-0.9518	−2.3606	−3.1678

**Table 4:** MLEs, Standard deviations, Bias, and MSE for some various values of parameters.

Sample Size	Actual Values			Estimated Values			Standard Deviations			Bias			Mean Square Error		
n	α	λ	a	$\hat{\alpha }$	$\hat{\lambda }$	$\hat{a}$	$\mathrm{sd}\left(\hat{\alpha }\right)$	$\mathrm{sd}\left(\hat{\lambda }\right)$	$\mathrm{sd}\left(\hat{a}\right)$	$\mathrm{Bias}\left(\hat{\alpha }\right)$	$\mathrm{Bias}\left(\hat{\lambda }\right)$	$\mathrm{Bias}\left(\hat{a}\right)$	$\mathrm{MSE}\left(\hat{\alpha }\right)$	$\mathrm{MSE}\left(\hat{\lambda }\right)$	$\mathrm{MSE}\left(\hat{a}\right)$
30	0.3	0.1	0.5	0.2930	0.7526	0.5822	0.0916	1.0682	0.1461	-0.0070	0.6526	0.0822	0.0084	1.5667	0.0281
	0.5	0.3	0.1	0.5721	0.4093	0.1125	0.2925	0.6730	0.0239	0.0721	0.1093	0.0125	0.0907	0.4649	0.0007
	0.1	0.8	0.6	0.1037	1.1375	0.6374	0.0364	1.6721	0.1497	0.0037	0.3375	0.0374	0.0013	2.9094	0.0238
	1.5	0.5	2.0	1.5008	0.8681	2.1868	0.3431	1.1441	0.6001	0.0008	0.3680	0.1868	0.1178	1.4444	0.3949
	0.2	2.0	0.2	0.3145	1.8311	0.1970	0.4751	1.6644	0.0510	0.1145	−0.1689	−0.0030	0.2388	2.7984	0.0026
	1.0	1.0	1.0	1.0260	1.1293	1.0201	0.3494	1.2945	0.2767	0.0260	0.1293	0.0202	0.1228	1.6924	0.0770
	0.1	2.5	0.8	0.1334	1.8896	0.7911	0.0580	1.6869	0.1676	0.0334	−0.6104	−0.0089	0.0045	3.2178	0.0282
	0.1	2.3	0.87	0.1286	1.7516	0.8644	0.0517	1.5180	0.1894	0.0286	−0.5484	−0.0057	0.0035	2.6050	0.0359
	1.3	1.1	1.5	1.3943	1.1345	1.5909	0.3892	1.2700	0.4059	0.0943	0.0345	0.0909	0.1604	1.6139	0.1730
50	0.3	0.1	0.5	0.2892	0.6294	0.5612	0.0713	0.9262	0.1138	−0.0108	0.5294	0.0612	0.0052	1.1380	0.0167
	0.5	0.3	0.1	0.5247	0.2621	0.1064	0.1275	0.4667	0.0161	0.0248	−0.0379	0.0064	0.0169	0.2192	0.0003
	0.1	0.8	0.6	0.1005	1.0509	0.6231	0.0288	1.1500	0.01196	0.0005	0.2509	0.0231	0.0008	1.3854	0.0148
	1.5	0.5	2.0	1.4437	0.7400	1.9299	0.2799	1.1384	0.4897	−0.0563	0.2400	−0.0702	0.0815	1.3535	0.2447
	0.2	2.0	0.2	0.2501	1.7826	0.1928	0.1759	1.2446	0.0458	0.0501	−0.2174	−0.0072	0.0335	1.5961	0.0025
	1.0	1.0	1.0	0.9985	1.1944	1.0062	0.2930	1.3341	0.2067	−0.0015	0.1944	0.0062	0.0858	1.8175	0.0428
	0.1	2.5	0.8	0.1219	2.0981	0.7828	0.0477	1.4728	0.1315	0.0219	−0.4019	−0.0172	0.0028	2.3305	0.0178
	0.1	2.3	0.87	0.1187	1.9504	0.8547	0.0432	1.4612	0.1455	0.019	0.3496	−0.0150	0.0022	2.2570	0.0214
	1.3	1.1	1.5	1.3308	1.2346	1.5304	0.3369	1.2361	0.2903	0.0308	0.0346	0.0304	0.1145	1.6030	0.0852
100	0.3	0.1	0.5	0.2895	0.4806	0.5405	0.0513	0.7105	0.0815	−0.0105	0.3806	0.0405	0.0028	0.6496	0.0083
	0.5	0.3	0.1	0.5033	0.3269	0.1015	0.0303	0.1670	0.0070	0.0036	0.0269	0.0015	$9.29\times 10^{-4}$	$2.86\times 10^{-2}$	$5.19\times 10^{-5}$
	0.1	0.8	0.6	0.0994	0.9789	0.6123	0.0222	1.0996	0.0911	−0.0007	0.1789	0.0122	0.0005	1.2410	0.0085
	1.5	0.5	2.0	1.4293	0.6856	1.8640	0.2415	1.1216	0.4095	−0.0708	0.1856	−0.1360	0.0633	1.2924	0.1861
	0.2	2.0	0.2	0.1996	1.7591	0.1865	0.1066	1.0954	0.0450	−0.0004	−0.2410	−0.0135	0.0114	1.2579	0.0022
	1.0	1.0	1.0	0.9889	1.2083	1.0038	0.2360	1.2935	0.1459	−0.0111	0.2083	0.0038	0.0558	1.7163	0.02129
	0.1	2.5	0.8	0.1115	2.3604	0.7848	0.0384	1.4364	0.0975	0.0115	−0.1396	−0.0152	0.0016	2.0826	0.0097
	0.1	2.3	0.87	0.1096	2.1827	0.8531	0.0354	1.4344	0.1080	0.0096	−0.1173	−0.0169	0.0014	2.0711	0.0120
	1.3	1.1	1.5	1.2967	1.2688	1.4863	0.2938	1.2132	0.2031	0.0034	0.0314	−0.0137	0.0863	1.5436	0.0415
150	0.3	0.1	0.5	0.2914	0.3870	0.5303	0.0409	0.5374	0.0664	−0.0086	0.2870	0.0303	0.0017	0.3711	0.0053
	0.5	0.3	0.1	0.5006	0.3066	0.1004	0.0119	0.0791	0.0033	0.0007	0.0066	0.0004	$1.41\times 10^{-4}$	$6.3\times 10^{-3}$	$1.1\times 10^{-5}$
	0.1	0.8	0.6	0.0994	0.9245	0.6074	0.0191	0.8763	0.0779	−0.0006	0.1245	0.0074	0.0004	0.7834	0.0061
	1.5	0.5	2.0	1.4334	0.6147	1.8281	0.2198	1.0580	0.3939	−0.0667	0.1147	−0.1719	0.0527	1.1324	0.1847
	0.2	2.0	0.2	0.1803	1.6778	0.1807	0.0907	1.0959	0.0476	−0.0197	−0.3223	−0.0193	0.0086	1.2048	0.0026
	1.0	1.0	1.0	0.9805	1.2255	1.0027	0.2153	1.2483	0.1251	−0.0195	0.2255	0.0027	0.0467	1.6090	0.0157
	0.1	2.5	0.8	0.1070	2.4901	0.7878	0.0344	1.4110	0.0794	0.0070	-0.0099	−0.0122	0.0012	1.9907	0.0065
	0.1	2.3	0.87	0.1059	2.3018	0.8557	0.0321	1.4126	0.0890	0.0059	0.0018	−0.0143	0.0011	1.9952	0.0081
	1.3	1.1	1.5	1.2825	1.3056	1.4850	0.2729	1.2032	0.1710	−0.0175	0.0528	−0.0151	0.0748	1.4061	0.0295
200	0.1	0.3	0.5	0.2926	0.3463	0.5257	0.0357	0.4722	0.0581	−0.0074	0.2463	0.0257	0.0013	0.2836	0.0040
	0.5	0.3	0.1	0.5002	0.3009	0.1001	0.0065	0.0230	0.0013	$2.2\times 10^{-4}$	$8.5\times 10^{-4}$	$7.4\times 10^{-5}$	$4.2\times 10^{-5}$	$5.3\times 10^{-4}$	$1.7\times 10^{-6}$
	0.1	0.8	0.6	0.0999	0.8651	0.6034	0.0163	0.7471	0.0710	−0.0001	0.0651	0.0034	0.0003	0.5624	0.0051
	1.5	0.5	2.0	1.4406	0.5526	1.8095	0.2070	0.9963	0.3866	−0.595	0.0526	−0.1905	0.0464	0.9952	0.1838
	0.2	2.	0.2	0.1677	1.5567	0.1740	0.0834	1.1169	0.0500	−0.0323	−0.4433	-0.0260	0.0080	1.2039	0.0022
	1.0	1.0	1.0	0.9805	1.2255	1.0027	0.2153	1.2483	0.1251	−0.0195	0.2255	0.0027	0.0467	1.6090	0.0157
	0.1	2.5	0.8	0.1048	2.5536	0.7899	0.0317	1.3723	0.0695	0.0048	0.0536	−0.0101	0.0010	1.8859	0.0049
	0.1	2.3	0.87	0.1033	2.3845	0.8583	0.0298	1.3826	0.0778	0.0033	0.0845	−0.0117	0.0009	1.9323	0.0062
	1.3	1.1	1.5	1.2779	1.3112	1.4871	0.2589	1.2010	0.1519	−0.0221	0.0511	−0.0129	0.0675	1.4030	0.0232
300	0.1	0.3	0.5	0.2937	0.2869	0.5190	0.0286	0.3793	0.0471	−0.0063	0.1869	0.0190	0.0009	0.1788	0.0026
	0.5	0.3	0.1	0.5001	0.3001	0.1000	0.0024	0.0067	0.005	$2.4\times 10^{-5}$	$1.2\times 10^{-4}$	$9.7\times 10^{-6}$	$5.7\times 10^{-6}$	$4.5\times 10^{-5}$	$2.4\times 10^{-7}$
	0.1	0.8	0.6	0.997	0.8449	0.6018	0.01365	0.6169	0.0602	−0.0003	0.0449	0.0018	0.0002	0.3825	0.0036
	1.5	0.5	2.0	1.4461	0.4892	1.7794	0.1903	0.9318	0.3850	−0.0539	−0.0108	−2206	0.0391	0.8684	0.1831
	0.2	2.0	0.2	0.2153	1.3274	0.2002	0.481	0.9477	0.0014	0.0153	−0.0726	0.0002	0.0025	1.2010	0.0002
	1.0	1.0	1.0	0.9901	1.1144	0.9978	0.1063	0.9928	0.0932	−0.0099	0.1144	−0.0022	0.0277	0.9986	0.0087
	1.2	2.5	0.8	0.1028	2.5891	0.7915	0.0281	1.3050	0.0571	0.0028	0.0891	−0.0085	0.0008	1.7107	0.0033
	0.1	2.3	0.87	0.1014	2.4315	0.8609	0.0266	1.3086	0.0635	0.0014	0.1315	−0.0091	0.0007	1.7296	0.0041
	1.3	1.1	1.5	1.2776	1.2838	1.4869	0.2352	1.1806	0.1268	−0.0224	0.1038	−0.0131	0.0558	1.3275	0.0162

**Table 5:** MLEs, R, $\hat{R}$, $Bias(\hat{R})$, $MSE(\hat{R})$, $Var(\hat{R})$, and ALCI for some parameter values.

(n, m)	$a_{1}$	$a_{2}$	λ	$\hat{a_{1}}$	$\hat{a_{2}}$	$\hat{\lambda }$	*R*	$\hat{R}$	$\mathrm{Bias}(\hat{R})$	$\mathrm{MSE}(\hat{R})$	$\mathrm{Var}(\hat{R})$	ALCI
(20, 20)	1.5	2.5	3.0	1.5734	2.6242	3.3101	0.3135	0.3155	−0.0018	0.0057	0.0449	0.8672
	1.5	0.5	1.9	1.6094	0.5387	2.1903	0.8040	0.8070	0.0030	0.0035	0.0230	0.5945
	0.9	0.5	2.0	0.9652	0.5362	2.3130	0.6841	0.6878	0.0036	0.0055	0.0446	0.8276
	0.8	1.7	2.5	0.8479	1.8004	2.7912	0.2565	0.2544	−0.0022	0.0047	0.0743	1.0682
	0.7	0.8	0.9	0.7808	0.8890	1.2374	0.4626	0.4636	−$7.54\times 10^{-5}$	0.0068	1.2532	4.3884
	1.7	2.8	3.5	1.7932	2.9562	3.8935	0.3081	0.3056	−0.0025	0.0057	0.0374	0.7581
	3.7	3.0	4.0	2.8006	2.3522	2.8152	0.5892	0.5577	−0.0316	0.0073	0.0518	0.8923
(30, 20)	1.5	2.5	3.0	1.5614	2.6288	3.2709	0.3153	0.3119	−0.0035	0.0048	0.0392	0.7759
	1.5	0.5	1.9	1.6137	0.5371	2.0804	0.8040	0.8051	0.0011	0.0034	0.0196	0.5480
	0.9	0.5	2.0	0.9469	0.5313	2.2317	0.6841	0.6852	0.0011	0.0045	0.0254	0.6254
	0.8	1.7	2.5	0.8371	1.7927	2.7557	0.2565	0.2529	−0.0036	0.0038	0.0372	0.7565
	0.7	0.8	0.9	0.7580	0.8779	1.1530	0.4626	0.4600	−0.0026	0.0055	0.8146	3.5379
	1.7	2.8	3.5	1.7736	2.9562	3.8935	0.3081	0.3061	−0.0021	0.0048	0.0339	0.7214
	3.7	3.0	4.0	3.0946	2.6644	3.3268	0.5892	0.5526	−0.0366	0.0082	0.0336	0.7187
(30, 40)	1.5	2.5	3.0	1.5230	2.5219	3.0900	0.3153	0.3177	0.0023	0.0039	0.0364	0.7481
	1.5	0.5	1.9	1.5792	0.5356	2.0192	0.8040	0.8015	−0.0025	0.0029	0.0099	0.3910
	0.9	0.5	2.0	0.9371	0.5190	2.1635	0.6841	0.6861	0.0019	0.0032	0.0049	0.2750
	0.8	1.7	2.5	0.8294	1.7507	2.6559	0.2565	0.2565	−$3.36\times 10^{-5}$	0.0027	0.0245	0.6131
	0.7	0.8	0.9	0.7284	0.8319	1.0114	0.4626	0.4624	−0.0002	0.0025	0.0357	0.7411
	1.7	2.8	3.5	1.7553	2.8778	3.7114	0.3081	0.3080	−0.0002	0.0034	0.0290	0.6670
	3.7	3.0	4.0	3.7540	3.0374	4.1474	0.5892	0.5898	0.0006	0.0041	0.0189	0.5387
(50, 50)	1.5	2.5	3.0	1.5341	2.5532	3.1299	0.3153	0.3149	−0.0005	0.0022	0.0159	0.4957
	1.5	0.5	1.9	1.5640	0.5178	1.9527	0.8040	0.8038	−0.0002	0.0019	0.0042	0.2543
	0.9	0.5	2.0	0.9237	0.5130	2.1048	0.6841	0.6853	0.0012	0.0022	0.0016	0.1541
	0.8	1.7	2.5	0.8212	1.7449	2.6221	0.2565	0.2552	−0.0013	0.0018	0.0098	0.3883
	0.7	0.8	0.9	0.7458	0.8512	1.0814	0.4626	0.4615	−0.0011	0.0038	0.0303	0.6825
	1.7	2.8	3.5	1.7370	2.8558	3.6418	0.3081	0.3076	−0.0005	0.0023	0.0119	0.4270
	3.7	3.0	4.0	3.7245	3.0374	4.0996	0.5892	0.5858	−0.0035	0.0034	0.0155	0.4885

**Table 6:** MLEs, log-likelihood, *ℓ*, AIC, BIC, CAIC, KS, AD, and CvM, (*p*-value in parenthesis) of the competing distributions for the first data set.

Model	α	β	θ	λ	*a*	*b*	*c*	L	AIC	BIC	CAIC	KS	AD	CvM
PoiGHL	0.0718	-	-	3.9829	1.2157	-	-	-410.72	827.43	835.99	827.62	0.0485	0.3528	0.0603
												(0.9243)	(0.4609)	(0.3721)
McHL	0.3582	-	-	-	0.3586	0.3329	3.4083	−412.37	832.75	844.16	833.07	0.0623	0.5438	0.0895
												(0.7029)	(0.1594)	(0.1546)
BHL	$5.81\times 10^{-3}$	-	-	-	1.1110	39.3700	-	−414.19	834.38	842.94	834.58	0.0779	0.8202	0.1372
												(0.4190)	(0.0332)	(0.0347)
KwHL	0.3759	-	-	-	1.0149	0.3362	-	−413.57	833.15	841.70	833.34	0.0833	0.7050	0.1179
												(0.3379)	(0.0642)	(0.0633)
THLBx	$1.06\times 10^{-3}$	-	0.4373	81.6600	-	-	-	−415.48	836.96	845.52	837.15	0.0768	1.0172	0.1730
												(0.4365)	(0.0108)	(0.0117)
GHL	0.1440	-	-	-	0.9527	-	-	−416.64	837.27	842.9755	837.37	0.0949	1.2663	0.2163
												(0.1994)	(0.0026)	(0.0033)
PHL	0.1479	-	-	$2.483\times 10^{-5}$	-	-	-	−416.73	837.45	843.16	837.55	0.0989	1.2406	0.2119
												(0.1631)	(0.0030)	(0.0037)
PwHL	0.8880	0.2015	-	-	-	-	-	−415.10	834.19	839.90	834.29	0.0765	0.9722	0.1654
												(0.4420)	(0.0140)	(0.0147)
HLP	0.0313	-	-	7.1580	-	-	-	−413.33	830.66	836.37	830.76	0.0930	0.4559	0.0763
												(0.2188)	(0.2632)	(0.2297)
HL	0.1479	-	-	-	-	-	-	−416.73	835.45	838.30	835.48	0.0989	1.2406	0.2119
												(0.1631)	(0.0030)	(0.0037)

**Table 7:** MLEs, log-likelihood, *ℓ*, AIC, BIC, CAIC, KS, AD, and CvM, (*p*-value in parenthesis) of the competing distributions for the second data set.

Model	α	β	θ	λ	*a*	*b*	L	AIC	BIC	CAIC	KS	AD	CvM
PoiGHL	19.0226	-	-	4.4492	6.6818	-	35.41	−64.82	−61.84	−63.32	0.1370	0.7601	0.1040
											(0.7990)	(0.0399)	(0.0914)
POGE-HL	40.9400	10.3810	-	1.3350	-	-	32.92	−59.84	−56.86	−58.34	0.1497	1.2059	0.1718
											(0.7247)	(0.0029)	(0.0109)
TIHLBx	0.4710	-	0.6786	64.1814	-	-	26.06	−46.11	−43.12	−44.61	0.2876	2.3938	0.3965
											(0.0587)	($2.64\times 10^{-6}$)	($2.29\times 10^{-5}$)
BHL	0.5680	-	-	-	3.8541	106.9291	29.50	−53.01	−50.02	−51.51	0.2338	1.9006	0.2987
											(0.1911)	($4.76\times 10^{-5}$)	(0.00028)
EGSHL	-	-	-	-	50.2194	11.4110	32.24	−60.47	−58.48	−59.77	0.1719	1.3311	0.1941
											(0.5391)	(0.0014)	(0.0056)
HLP	1.0000	-	-	16.5311	-	-	22.13	−40.26	−38.27	−39.56	0.4251	1.7893	0.2776
											(0.00085)	($9.17\times 10^{-5}$)	(0.0005)
GHL	29.1899	-	-	-	8.2315	-	32.64	−61.289	−59.30	−60.58	0.1607	1.2237	0.1757
											(0.6236)	(0.0026)	(0.0097)
PHL	12.4294	-	-	$8.47\times 10^{-5}$	-	-	24.34	−44.69	−42.69	−43.98	0.3939	1.9953	0.3164
											(0.0026)	($2.8\times 10^{-5}$)	(0.00018)
OGHL	24.7668	0.4163	-	-	-	-	24.97	−45.95	−43.95	−45.24	0.3780	1.7672	0.2734
											(0.0045)	(0.0001)	(0.00057)
HL	12.4291	-	-	-	-	-	24.34	−46.69	−45.69	−46.46	0.3939	1.9913	0.3164
											(0.00026)	$(2.79\times 10^{-5}$)	(0.00018)

**Table 8:** MLEs, log-likelihood, *ℓ*, AIC, BIC, CAIC, KS, AD, and CvM, (*p*-value in parenthesis) of the competing distributions for the third data set.

Model	α	β	θ	λ	*a*	*b*	*c*	*ℓ*	AIC	BIC	CAIC	KS	AD	CvM
PoiGHL	0.119	-	-	5.0802	0.6944	-	-	−135.29	276.57	281.15	277.37	0.2065	1.4015	0.2490
												(0.0950)	(0.0011)	(0.0012)
McHL	o.1193	-	-	-	1.5867	0.3148	0.2119	−144.71	297.42	303.52	298.80	0.3342	2.8523	0.5377
												(0.0007)	($2.45\times 10^{-7}$)	($1.06\times 10^{-6}$)
TIHLBx	$8.72\times 10^{-5}$	-	0.2642	40.6600	-	-	-	−136.66	279.32	283.90	280.12	0.2075	1.5922	0.2866
												(0.0923)	(0.00035)	(0.00043)
KwHL	0.1172	-	-	-	0.4077	0.3117	-	−144.41	294.83	299.41	295.63	0.3539	2.7622	0.5196
												(0.0003)	($4.1\times 10^{-7}$)	($1.55\times 10^{-6}$)
BHL	0.1109	-	-	-	0.3087	0.3239	-	−144.93	295.85	300.43	296.65	0.2291	2.9305	0.5535
												(0.0474)	($1.56\times 10^{-7}$)	($7.74\times 10^{-7}$)
GHLP	0.0176	-	-	4.2976	0.6561	-	-	−137.03	280.06	284.64	280.86	0.2449	1.7197	0.3109
												(0.0279)	(0.00016)	(0.00022)
EGSHL	-	-	-	-	0.0238	0.4836	-	−137.45	278.90	281.96	279.29	0.2672	1.7163	0.31176
												(0.0124)	(0.00017)	(0.0022)
HLP	0.0156	-	-	6.6167	-	-	-	−139.27	282.53	285.58	282.92	0.3004	1.6233	0.2902
												(0.0032)	(0.0003)	(0.0004)
GHL	0.0275	-	-	-	0.4346	-	-	−141.46	286.93	289.98	287.31	0.3094	2.4062	0.4473
												(0.0022)	($3.18\times 10^{-6}$)	($7.6\times 10^{-5}$)
PHL	0.0048	-	-	$4.42\times 10^{-8}$	-	-	-	−151.80	307.60	310.65	307.99	0.4748	2.4034	0.4471
												($1.35\times 10^{-7}$)	($3.24\times 10^{-6}$)	($7.62\times 10^{-6}$)
OGH	0.0496	0.9976	-	-	-	-	-	−151.80	307.60	310.65	307.99	0.4758	2.4053	0.4471
												($1.36\times 10^{-7}$)	($3.23\times 10^{-6}$)	($7.63\times 10^{-6}$)
HL	0.0496	-	-	-	-	-	-	−151.80	305.60	307.12	305.72	0.4759	2.4034	0.4471
												($1.35\times 10^{-7}$)	($3.23\times 10^{-6}$)	($7.63\times 10^{-6}$)

**Table 9:** LR- statistic and *p*-value for the first, second and third data sets.

$H_{0}:a=1$, HLP vs. $H_{1}:a\neq 1$, PoiGHL			
	**First Data**	**Second Data**	**Third Data**
LR-statistic	5.22	26.56	7.96
*p*-value	0.0223	$2.55\times 10^{-7}$	0.0048

## Appendix A. Proofs of Theorems 2 and 3

### Appendix A.1. Proof of Theorem 2

Sufficiently, let $Q\left(x\right)=e^{-\lambda \left(\frac{1-e^{-\alpha x}}{1+e^{-\alpha x}}\right)}$, then

$$\begin{array}{cc} E\left[e^{-\lambda \left(\frac{1-e^{-\alpha X}}{1+e^{-\alpha X}}\right)}|X\geq x\right] & =\frac{1}{1-F(x)}\int_{x}^{\infty }e^{-\lambda \left(\frac{1-e^{-\alpha y}}{1+e^{-\alpha y}}\right)}f\left(y\right)dy\\ & =\frac{h\left(x\right)\;e^{\lambda \left(\frac{1-e^{-\alpha x}}{1+e^{-\alpha x}}\right)}}{\frac{e^{-\alpha x}}{{\left(1+e^{-\alpha x}\right)}^{2}}}\int_{x}^{\infty }\frac{e^{-\alpha y}}{{\left(1+e^{-\alpha y}\right)}^{2}}e^{-2\lambda \left(\frac{1-e^{-\alpha y}}{1+e^{-\alpha y}}\right)}dy, \end{array}$$

by letting $u=e^{-\lambda \left(\frac{1-e^{-\alpha y}}{1+e^{-\alpha y}}\right)}$ we have

$$\begin{array}{cc} E\left[e^{-\lambda \left(\frac{1-e^{-\alpha X}}{1+e^{-\alpha X}}\right)}|X\geq x\right] & =\frac{h\left(x\right)\;e^{\lambda \left(\frac{1-e^{-\alpha x}}{1+e^{-\alpha x}}\right)}}{2\lambda \alpha \frac{e^{-\alpha x}}{{\left(1+e^{-\alpha x}\right)}^{2}}}\int_{e^{-\lambda }}^{e^{-\lambda \left(\frac{1-e^{-\alpha x}}{1+e^{-\alpha x}}\right)}}u\;du, \end{array}$$

thus,

$$E\left[e^{-\lambda \left(\frac{1-e^{-\alpha X}}{1+e^{-\alpha X}}\right)}|X\geq x\right]=h\left(x\right)\Psi \left(x\right),$$

where $\Psi \left(x\right)=\frac{\left[e^{-2\lambda \left(\frac{1-e^{-\alpha x}}{1+e^{-\alpha x}}\right)}-e^{-2\lambda }\right]e^{\lambda \left(\frac{1-e^{-\alpha x}}{1+e^{-\alpha x}}\right)}}{4\alpha \;\lambda \;\frac{e^{-\alpha x}}{{\left(1+e^{-\alpha x}\right)}^{2}}}$. Next, we obtain $\frac{Q(x)}{\Psi (x)}$ and $\frac{\Psi^{\prime }\left(x\right)}{\Psi (x)}$ as

$$\frac{Q(x)}{\Psi (x)}=\frac{4\alpha \;\lambda \;\frac{e^{-\alpha x}}{{\left(1+e^{-\alpha x}\right)}^{2}}e^{-2\lambda \left(\frac{1-e^{-\alpha x}}{1+e^{-\alpha x}}\right)}}{\left[e^{-2\lambda \left(\frac{1-e^{-\alpha x}}{1+e^{-\alpha x}}\right)}-e^{-2\lambda }\right]},$$

$$\frac{\Psi^{\prime }\left(x\right)}{\Psi (x)}=-\frac{4\alpha \lambda \frac{e^{-\alpha x}}{{\left(1+e^{-\alpha x}\right)}^{2}}e^{-2\lambda \left(\frac{1-e^{-\alpha x}}{1+e^{-\alpha x}}\right)}}{\left[e^{-2\lambda \left(\frac{1-e^{-\alpha x}}{1+e^{-\alpha x}}\right)}-e^{-2\lambda }\right]}+\alpha -\frac{2\alpha e^{-\alpha x}}{\left(1+e^{-\alpha x}\right)}+\frac{2\lambda \alpha e^{-\alpha x}}{{\left(1+e^{-\alpha x}\right)}^{2}}.$$

This implies that

$$\frac{Q\left(x\right)+\Psi^{\prime }\left(x\right)}{\Psi (x)}=\alpha -\frac{2\alpha e^{-\alpha x}}{\left(1+e^{-\alpha x}\right)}+\frac{2\lambda \alpha e^{-\alpha x}}{{\left(1+e^{-\alpha x}\right)}^{2}},$$

hence for all x>0,

$$\int_{0}^{x}\frac{Q\left(y\right)+\Psi^{\prime }\left(y\right)}{\Psi (y)}dy=\alpha x+2\log\left(1+e^{-\alpha x}\right)+\lambda \left(\frac{1-e^{-\alpha x}}{1+e^{-\alpha x}}\right)-\log4,$$

thus, by Lemma 2 and for all x>0 we get

$$f\left(x\right)=\Lambda exp\left[-\int_{0}^{x}\frac{Q\left(y\right)+\Psi^{\prime }\left(y\right)}{\Psi (y)}dy\right]=\frac{4\Lambda e^{-\alpha x}}{\;{\left(1+e^{-\alpha x}\right)}^{2}}e^{-\lambda \left(\frac{1-e^{-\alpha x}}{1+e^{-\alpha x}}\right)},$$

with normalizing constant $\Lambda =\frac{\alpha \;\lambda }{2(1-e^{-\lambda })}$.

### Appendix A.2. Proof of Theorem 3

Sufficiently, let $Q\left(x\right)=e^{-\lambda \left(\frac{1-e^{-\alpha x}}{1+e^{-\alpha x}}\right)}$, then, in similar way,

$$\begin{array}{cc} E\left[e^{-\lambda \left(\frac{1-e^{-\alpha X}}{1+e^{-\alpha X}}\right)}|X\leq x\right] & =\frac{1}{F(x)}\;\int_{0}^{x}e^{-\lambda \left(\frac{1-e^{-\alpha y}}{1+e^{-\alpha y}}\right)}f\left(y\right)dy\\ & =\frac{r(x)}{f(x)}\;\int_{0}^{x}e^{-\lambda \left(\frac{1-e^{-\alpha y}}{1+e^{-\alpha y}}\right)}f\left(y\right)dy, \end{array}$$

by some algebra, and letting $u=e^{-\lambda \left(\frac{1-e^{-\alpha y}}{1+e^{-\alpha y}}\right)}$ we get

$$\begin{array}{cc} E\left[e^{-\lambda \left(\frac{1-e^{-\alpha X}}{1+e^{-\alpha X}}\right)}|X\leq x\right] & =-\frac{r\left(x\right)\;e^{\lambda \left(\frac{1-e^{-\alpha x}}{1+e^{-\alpha x}}\right)}}{2\lambda \alpha \frac{e^{-\alpha x}}{{\left(1+e^{-\alpha x}\right)}^{2}}}\int_{1}^{e^{-\lambda \left(\frac{1-e^{-\alpha x}}{1+e^{-\alpha x}}\right)}}u\;du=r\left(x\right)V\left(x\right), \end{array}$$

where

$$V\left(x\right)=\frac{\left[1-e^{-2\lambda \left(\frac{1-e^{-\alpha x}}{1+e^{-\alpha x}}\right)}\right]e^{\lambda \left(\frac{1-e^{-\alpha x}}{1+e^{-\alpha x}}\right)}}{4\alpha \;\lambda \;\frac{e^{-\alpha x}}{{\left(1+e^{-\alpha x}\right)}^{2}}}.$$

Then we compute,

$$\frac{Q(x)}{V(x)}=\frac{4\alpha \;\lambda \;\frac{e^{-\alpha x}}{{\left(1+e^{-\alpha x}\right)}^{2}}e^{-2\lambda \left(\frac{1-e^{-\alpha x}}{1+e^{-\alpha x}}\right)}}{\left[1-e^{-2\lambda \left(\frac{1-e^{-\alpha x}}{1+e^{-\alpha x}}\right)}\right]}$$

and

$$\frac{V^{\prime }\left(x\right)}{V(x)}=-\frac{4\alpha \;\lambda \;\frac{e^{-\alpha x}}{{\left(1+e^{-\alpha x}\right)}^{2}}e^{-2\lambda \left(\frac{1-e^{-\alpha x}}{1+e^{-\alpha x}}\right)}}{\left[1-e^{-2\lambda \left(\frac{1-e^{-\alpha x}}{1+e^{-\alpha x}}\right)}\right]}+\alpha -\frac{2\alpha e^{-\alpha x}}{1+e^{-\alpha x}}+\frac{2\alpha \lambda e^{-\alpha x}}{{\left(1+e^{-\alpha x}\right)}^{2}},$$

this implies that

$$\frac{V^{\prime }\left(x\right)-Q\left(x\right)}{V(x)}=\alpha -\frac{2\alpha e^{-\alpha x}}{1+e^{-\alpha x}}+\frac{2\alpha \lambda e^{-\alpha x}}{{\left(1+e^{-\alpha x}\right)}^{2}}$$

and

$$\int_{0}^{x}\frac{Q\left(y\right)+\Psi^{\prime }\left(y\right)}{\Psi (y)}dy=\alpha x+2\log\left(1+e^{-\alpha x}\right)+\lambda \left(\frac{1-e^{-\alpha x}}{1+e^{-\alpha x}}\right)-\log4,\;\;x>0.$$

By considering the Lemma 3 We Obtain

$$f\left(x\right)=\Lambda \exp\left[-\int_{0}^{x}\frac{V^{\prime }\left(y\right)-Q\left(y\right)}{V(y)}dy\right]=\frac{4\Lambda e^{-\alpha x}}{\;{\left(1+e^{-\alpha x}\right)}^{2}}e^{-\lambda \left(\frac{1-e^{-\alpha x}}{1+e^{-\alpha x}}\right)},\;\;x>0,$$

where the normalizing constant $\Lambda =\frac{\alpha \;\lambda }{2(1-e^{-\lambda })}$.

## Appendix B. The Elements of Information Matrix

$$\begin{array}{cc} J_{\alpha \alpha } & =-\frac{n}{\alpha^{2}}-\left(a+1\right)\sum_{i=1}^{n}\frac{x_{i}^{2}e^{-\alpha x_{i}}}{{\bar{\nu }}_{i}}+\left(a+1\right)\sum_{i=1}^{n}\frac{x_{i}^{2}e^{-2\alpha x_{i}}}{{\bar{\nu }}_{i}^{2}}-\left(a-1\right)\sum_{i=1}^{n}\frac{x_{i}^{2}e^{-\alpha x_{i}}}{\nu_{i}}\\ & -\left(a-1\right)\sum_{i=1}^{n}\frac{x_{i}^{2}e^{-2\alpha x_{i}}}{\nu_{i}^{2}}+2a\lambda \sum_{i=1}^{n}\frac{x_{i}^{2}e^{-\alpha x_{i}}\;\nu_{i}^{a-1}}{{\bar{\nu }}_{i}^{a+1}}\\ & -4a\lambda \left(a-1\right)\sum_{i=1}^{n}\frac{x_{i}^{2}e^{-2\alpha x_{i}}\;\nu_{i}^{a-2}}{{\bar{\nu }}_{i}^{a+2}}-4a\lambda \sum_{i=1}^{n}\frac{x_{i}^{2}e^{-2\alpha x_{i}}\;\nu_{i}^{a-1}}{{\bar{\nu }}_{i}^{a+2}}\\ J_{aa} & =-\frac{n}{a^{2}}-\lambda \sum_{i=1}^{n}{\left(\frac{\nu_{i}}{{\bar{\nu }}_{i}}\right)}^{a}\log^{2}\left(\frac{\nu_{i}}{{\bar{\nu }}_{i}}\right),\;\;\;\;J_{\lambda \lambda }=-\frac{n}{\lambda^{2}}+\frac{ne^{-\lambda }}{{\left(1-e^{-\lambda }\right)}^{2}},\\ J_{\alpha \lambda } & =-a\sum_{i=1}^{n}{\left(\frac{\nu_{i}}{{\bar{\nu }}_{i}}\right)}^{a-1}\frac{2x_{i}e^{-\alpha x_{i}}}{{\bar{\nu }}_{i}^{2}},\;\;\;J_{a\lambda }=-\sum_{i=1}^{n}{\left(\frac{\nu_{i}}{{\bar{\nu }}_{i}}\right)}^{a}\log\left(\frac{\nu_{i}}{{\bar{\nu }}_{i}}\right),\\ J_{\alpha a} & =\sum_{i=1}^{n}\frac{x_{i}e^{-\alpha x_{i}}}{{\bar{\nu }}_{i}}+\sum_{i=1}^{n}\frac{x_{i}e^{-\alpha x_{i}}}{\nu_{i}}-2\lambda \sum_{i=1}^{n}{\left(\frac{\nu_{i}}{{\bar{\nu }}_{i}}\right)}^{a-1}\frac{x_{i}e^{-\alpha x_{i}}}{{\bar{\nu }}_{i}^{2}},\\ & -2a\lambda \sum_{i=1}^{n}{\left(\frac{\nu_{i}}{{\bar{\nu }}_{i}}\right)}^{a-1}\frac{x_{i}e^{-\alpha x_{i}}}{{\bar{\nu }}_{i}^{2}}\log\left(\frac{\nu_{i}}{{\bar{\nu }}_{i}}\right), \end{array}$$

where $\nu_{i}=1-e^{-\alpha x_{i}}$ and ${\bar{\nu }}_{i}=1+e^{-\alpha x_{i}}$.

## Appendix C. Element of Information Matrix for the Estimators of R, Computations of the *u_ij_* and $\frac{\partial R}{\partial a_{1}}$, $\frac{\partial R}{\partial a_{1}}$ and $\frac{\partial R}{\partial \lambda }$

### Appendix C.1. Element of Information Matrix for the Estimators of R

$$\begin{array}{cc} \frac{\partial^{2}\ell }{\partial a_{1}^{2}} & =-\frac{n}{a_{1}^{2}}-\lambda \sum_{i=1}^{n}{\left(\frac{1-e^{-x}}{1+e^{-x}}\right)}^{a_{1}}\log^{2}\left(\frac{1-e^{-x}}{1+e^{-x}}\right)\\ \frac{\partial^{2}\ell }{\partial a_{2}^{2}}= & -\frac{m}{a_{2}^{2}}-\lambda \sum_{j=1}^{m}{\left(\frac{1-e^{-y}}{1+e^{-y}}\right)}^{a_{2}}\log^{2}\left(\frac{1-e^{-y}}{1+e^{-y}}\right)\\ \frac{\partial^{2}\ell }{\partial a_{1}\partial \lambda } & =-\sum_{i=1}^{n}{\left(\frac{1-e^{-x}}{1+e^{-x}}\right)}^{a_{1}}\log\left(\frac{1-e^{-x}}{1+e^{-x}}\right)\\ \frac{\partial^{2}\ell }{\partial a_{2}\partial \lambda } & =-\sum_{j=1}^{m}{\left(\frac{1-e^{-y}}{1+e^{-y}}\right)}^{a_{2}}\log\left(\frac{1-e^{-y}}{1+e^{-y}}\right)\\ \frac{\partial^{2}\ell }{\partial a_{2}\partial a_{1}} & =\frac{\partial^{2}\ell }{\partial a_{1}\partial a_{2}}=0\\ \frac{\partial^{2}\ell }{\partial \lambda^{2}}= & -\frac{\left(n+m\right)}{\lambda }+\frac{\left(n+m\right)e^{-\lambda }}{{\left(1-e^{-\lambda }\right)}^{2}} \end{array}$$

### Appendix C.2. Computations of the u_ij_

$$\begin{array}{cc} u_{11}=E\left[\frac{\partial^{2}\ell }{\partial a_{1}^{2}}\right] & =-\frac{n}{a_{1}^{2}}-\lambda \sum_{i=1}^{n}E\left[{\left(\frac{1-e^{-x}}{1+e^{-x}}\right)}^{a_{1}}\log^{2}\left(\frac{1-e^{-x}}{1+e^{-x}}\right)\right]\\ & =-\frac{n}{a_{1}^{2}}-\lambda \sum_{i=1}^{n}\frac{\partial^{2}}{\partial t^{2}}E{\left[{\left(\frac{1-e^{-x}}{1+e^{-x}}\right)}^{a_{1}+t}\right]}_{t=0}\\ & =-\frac{n}{a_{1}^{2}}-\lambda \sum_{i=1}^{n}\frac{\partial^{2}}{\partial t^{2}}\;\int_{0}^{\infty }{\left(\frac{1-e^{-x}}{1+e^{-x}}\right)}^{a_{1}+t}{f\left(x\right)dx\;|}_{t=0}\\ & =-\frac{n}{a_{1}^{2}}-\frac{2a_{1}\lambda^{2}n}{\left(1-e^{-\lambda }\right)}\frac{\partial^{2}}{\partial t^{2}}A^{*}\left(2a_{1}+t-1,2a_{1}+t+1,a_{1}\right){\;|}_{t=0}\\ u_{13}=E\left[\frac{\partial^{2}\ell }{\partial a_{1}\partial \lambda }\right] & =-\sum_{i=1}^{n}E\left[{\left(\frac{1-e^{-x}}{1+e^{-x}}\right)}^{a_{1}}\log\left(\frac{1-e^{-x}}{1+e^{-x}}\right)\right]\\ & =-\sum_{i=1}^{n}\frac{\partial }{\partial t}E{\left[{\left(\frac{1-e^{-x}}{1+e^{-x}}\right)}^{a_{1}+t}\right]}_{t=0}\\ & =-\sum_{i=1}^{n}\frac{\partial }{\partial t}\;\int_{0}^{\infty }{\left(\frac{1-e^{-x}}{1+e^{-x}}\right)}^{a_{1}+t}{f\left(x\right)dx\;|}_{t=0}\\ & =-\frac{2a_{1}\lambda n}{\left(1-e^{-\lambda }\right)}\frac{\partial }{\partial t}A^{*}\left(2a_{1}+t-1,2a_{1}+t+1,a_{1}\right){\;|}_{t=0}. \end{array}$$

In similar way,

$$\begin{array}{cc} u_{22}=E\left[\frac{\partial^{2}\ell }{\partial a_{2}^{2}}\right] & =-\frac{m}{a_{2}^{2}}-\lambda \sum_{j=1}^{m}E\left[{\left(\frac{1-e^{-y}}{1+e^{-y}}\right)}^{a_{2}}\log^{2}\left(\frac{1-e^{-y}}{1+e^{-y}}\right)\right]\\ & =-\frac{m}{a_{2}^{2}}-\frac{2a_{2}\lambda^{2}m}{\left(1-e^{-\lambda }\right)}\frac{\partial^{2}}{\partial t^{2}}A^{*}\left(2a_{2}+t-1,2a_{2}+t+1,a_{2}\right){\;|}_{t=0}\\ u_{23}=E\left[\frac{\partial^{2}\ell }{\partial a_{2}\partial \lambda }\right] & =-\sum_{j=1}^{m}E\left[{\left(\frac{1-e^{-y}}{1+e^{-y}}\right)}^{a_{2}}\log\left(\frac{1-e^{-y}}{1+e^{-y}}\right)\right]\\ & =-\sum_{j=1}^{m}\frac{\partial }{\partial t}E{\left[{\left(\frac{1-e^{-y}}{1+e^{-y}}\right)}^{a_{2}+t}\right]}_{t=0}\\ & =-\frac{2a_{2}\lambda m}{\left(1-e^{-\lambda }\right)}\frac{\partial }{\partial t}A^{*}\left(2a_{2}+t-1,2a_{2}+t+1,a_{2}\right){\;|}_{t=0}. \end{array}$$

### Appendix C.3. Computations of $\frac{\partial R}{\partial a_{1}}$, $\frac{\partial R}{\partial a_{1}}$ and $\frac{\partial R}{\partial \lambda }$

$$\begin{array}{cc} \frac{\partial R}{\partial a_{1}} & =-\frac{\partial }{\partial a_{1}}\int_{0}^{\infty }\frac{2a_{1}\lambda }{{\left(1-e^{-\lambda }\right)}^{2}}\frac{e^{-x}\;{\left(1-e^{-x}\right)}^{a_{1}-1}}{{\left(1+e^{-x}\right)}^{a_{1}+1}}e^{-\lambda {\left(\frac{1-e^{-x}}{1+e^{-x}}\right)}^{a_{1}}}e^{-\lambda {\left(\frac{1-e^{-x}}{1+e^{-x}}\right)}^{a_{2}}}dx\\ & =-\frac{2\lambda }{{\left(1-e^{-\lambda }\right)}^{2}}\int_{0}^{\infty }\frac{e^{-x}\;{\left(1-e^{-x}\right)}^{a_{1}-1}}{{\left(1+e^{-x}\right)}^{a_{1}+1}}e^{-\lambda {\left(\frac{1-e^{-x}}{1+e^{-x}}\right)}^{a_{1}}}e^{-\lambda {\left(\frac{1-e^{-x}}{1+e^{-x}}\right)}^{a_{2}}}dx\\ & -\frac{2a_{1}\lambda }{{\left(1-e^{-\lambda }\right)}^{2}}\int_{0}^{\infty }\frac{e^{-x}\;{\left(1-e^{-x}\right)}^{a_{1}-1}}{{\left(1+e^{-x}\right)}^{a_{1}+1}}\log\left(\frac{1-e^{-x}}{1+e^{-x}}\right)e^{-\lambda {\left(\frac{1-e^{-x}}{1+e^{-x}}\right)}^{a_{1}}}e^{-\lambda {\left(\frac{1-e^{-x}}{1+e^{-x}}\right)}^{a_{2}}}dx\\ & +\frac{2a_{1}\lambda^{2}}{{\left(1-e^{-\lambda }\right)}^{2}}\int_{0}^{\infty }\frac{e^{-x}\;{\left(1-e^{-x}\right)}^{a_{1}-1}}{{\left(1+e^{-x}\right)}^{a_{1}+1}}{\left(\frac{1-e^{-x}}{1+e^{-x}}\right)}^{a_{1}}\log\left(\frac{1-e^{-x}}{1+e^{-x}}\right)e^{-\lambda {\left(\frac{1-e^{-x}}{1+e^{-x}}\right)}^{a_{1}}}e^{-\lambda {\left(\frac{1-e^{-x}}{1+e^{-x}}\right)}^{a_{2}}}dx\\ & =-\frac{2\lambda }{{\left(1-e^{-\lambda }\right)}^{2}}\sum_{k=0}^{\infty }\frac{{\left(-\lambda \right)}^{k}}{k!}\int_{0}^{\infty }\frac{e^{-x}\;{\left(1-e^{-x}\right)}^{a_{1}+a_{2}k-1}}{{\left(1+e^{-x}\right)}^{a_{1}+a_{2}k+1}}e^{-\lambda {\left(\frac{1-e^{-x}}{1+e^{-x}}\right)}^{a_{1}}}dx\\ & -\frac{2a_{1}\lambda }{{\left(1-e^{-\lambda }\right)}^{2}}\sum_{k=0}^{\infty }\frac{{\left(-\lambda \right)}^{k}}{k!}\frac{\partial }{\partial t}\int_{0}^{\infty }\frac{e^{-x}\;{\left(1-e^{-x}\right)}^{a_{1}+a_{2}k+t-1}}{{\left(1+e^{-x}\right)}^{a_{1}+a_{2}k+t+1}}e^{-\lambda {\left(\frac{1-e^{-x}}{1+e^{-x}}\right)}^{a_{1}}}{dx|}_{t=0}\\ & +\frac{2a_{1}\lambda^{2}}{{\left(1-e^{-\lambda }\right)}^{2}}\sum_{k=0}^{\infty }\frac{\partial }{\partial t}\int_{0}^{\infty }\frac{e^{-x}\;{\left(1-e^{-x}\right)}^{2a_{1}+a_{2}k+t-1}}{{\left(1+e^{-x}\right)}^{a_{1}+a_{2}k+t+1}}e^{-\lambda {\left(\frac{1-e^{-x}}{1+e^{-x}}\right)}^{a_{1}}}{dx|}_{t=0}\\ & =-\frac{2\lambda }{{\left(1-e^{-\lambda }\right)}^{2}}\sum_{k=0}^{\infty }\frac{{\left(-\lambda \right)}^{k}}{k!}A^{*}\left(a_{1}+a_{2}k-1,a_{1}+a_{2}k+1,a_{1}\right)\\ & -\frac{2a_{1}\lambda }{{\left(1-e^{-\lambda }\right)}^{2}}\sum_{k=0}^{\infty }\frac{{\left(-\lambda \right)}^{k}}{k!}\frac{\partial }{\partial t}A^{*}\left(a_{1}+a_{2}k+t-1,a_{1}+a_{2}k+t+1,a_{1}\right){|}_{t=0}\\ & +\frac{2a_{1}\lambda^{2}}{{\left(1-e^{-\lambda }\right)}^{2}}\sum_{k=0}^{\infty }\frac{\partial }{\partial t}A^{*}\left(2a_{1}+a_{2}k+t-1,2a_{1}+a_{2}k+t+1,a_{1}\right){|}_{t=0} \end{array}$$

$$\begin{array}{cc} \frac{\partial R}{\partial a_{2}} & =-\frac{2a_{1}\lambda }{{\left(1-e^{-\lambda }\right)}^{2}}\;\frac{\partial }{\partial a_{2}}\;\int_{0}^{\infty }\frac{e^{-x}\;{\left(1-e^{-x}\right)}^{a_{1}-1}}{{\left(1+e^{-x}\right)}^{a_{1}+1}}e^{-\lambda {\left(\frac{1-e^{-x}}{1+e^{-x}}\right)}^{a_{1}}}e^{-\lambda {\left(\frac{1-e^{-x}}{1+e^{-x}}\right)}^{a_{2}}}dx\\ & =\frac{2a_{1}\lambda^{2}}{{\left(1-e^{-\lambda }\right)}^{2}}\int_{0}^{\infty }\frac{e^{-x}\;{\left(1-e^{-x}\right)}^{a_{1}-1}}{{\left(1+e^{-x}\right)}^{a_{1}+1}}{\left(\frac{1-e^{-x}}{1+e^{-x}}\right)}^{a_{2}}\log\left(\frac{1-e^{-x}}{1+e^{-x}}\right)e^{-\lambda {\left(\frac{1-e^{-x}}{1+e^{-x}}\right)}^{a_{1}}}e^{-\lambda {\left(\frac{1-e^{-x}}{1+e^{-x}}\right)}^{a_{2}}}dx\\ & =\frac{2a_{1}\lambda^{2}}{{\left(1-e^{-\lambda }\right)}^{2}}\sum_{k=0}^{\infty }\frac{{\left(-\lambda \right)}^{k}}{k!}\frac{\partial }{\partial t}\;\int_{0}^{\infty }\frac{e^{-x}\;{\left(1-e^{-x}\right)}^{a_{1}\left(k+1\right)+a_{2}+t-1}}{{\left(1+e^{-x}\right)}^{a_{1}\left(k+1\right)+a_{2}+t+1}}e^{-\lambda {\left(\frac{1-e^{-x}}{1+e^{-x}}\right)}^{a_{2}}}{dx|}_{t=0}\\ & =\frac{2a_{1}\lambda^{2}}{{\left(1-e^{-\lambda }\right)}^{2}}\sum_{k=0}^{\infty }\frac{{\left(-\lambda \right)}^{k}}{k!}\frac{\partial }{\partial t}A^{*}\left(a_{1}\left(k+1\right)+a_{2}+t-1,a_{1}\left(k+1\right)+a_{2}+t+1,a_{2}\right){|}_{t=0} \end{array}$$

$$\begin{array}{cc} \frac{\partial R}{\partial \lambda } & =-\frac{e^{-\lambda }}{{\left(1-e^{-\lambda }\right)}^{2}}-\frac{\partial }{\partial \lambda }\int_{0}^{\infty }\frac{2a_{1}\lambda }{{\left(1-e^{-\lambda }\right)}^{2}}\frac{e^{-x}\;{\left(1-e^{-x}\right)}^{a_{1}-1}}{{\left(1+e^{-x}\right)}^{a_{1}+1}}e^{-\lambda {\left(\frac{1-e^{-x}}{1+e^{-x}}\right)}^{a_{1}}}e^{-\lambda {\left(\frac{1-e^{-x}}{1+e^{-x}}\right)}^{a_{2}}}dx\\ & =-\frac{e^{-\lambda }}{{\left(1-e^{-\lambda }\right)}^{2}}-\frac{2a_{1}}{{\left(1-e^{-\lambda }\right)}^{2}}\int_{0}^{\infty }\frac{e^{-x}\;{\left(1-e^{-x}\right)}^{a_{1}-1}}{{\left(1+e^{-x}\right)}^{a_{1}+1}}e^{-\lambda {\left(\frac{1-e^{-x}}{1+e^{-x}}\right)}^{a_{1}}}e^{-\lambda {\left(\frac{1-e^{-x}}{1+e^{-x}}\right)}^{a_{2}}}dx\\ & +\frac{4a_{1}\lambda e^{-\lambda }}{{\left(1-e^{-\lambda }\right)}^{3}}\int_{0}^{\infty }\frac{e^{-x}\;{\left(1-e^{-x}\right)}^{a_{1}-1}}{{\left(1+e^{-x}\right)}^{a_{1}+1}}e^{-\lambda {\left(\frac{1-e^{-x}}{1+e^{-x}}\right)}^{a_{1}}}e^{-\lambda {\left(\frac{1-e^{-x}}{1+e^{-x}}\right)}^{a_{2}}}dx\\ & +\frac{2a_{1}\lambda }{{\left(1-e^{-\lambda }\right)}^{2}}\int_{0}^{\infty }\frac{e^{-x}\;{\left(1-e^{-x}\right)}^{a_{1}-1}}{{\left(1+e^{-x}\right)}^{a_{1}+1}}{\left(\frac{1-e^{-x}}{1+e^{-x}}\right)}^{a_{1}}e^{-\lambda {\left(\frac{1-e^{-x}}{1+e^{-x}}\right)}^{a_{1}}}e^{-\lambda {\left(\frac{1-e^{-x}}{1+e^{-x}}\right)}^{a_{2}}}dx\\ & +\frac{2a_{1}\lambda }{{\left(1-e^{-\lambda }\right)}^{2}}\int_{0}^{\infty }\frac{e^{-x}\;{\left(1-e^{-x}\right)}^{a_{1}-1}}{{\left(1+e^{-x}\right)}^{a_{1}+1}}{\left(\frac{1-e^{-x}}{1+e^{-x}}\right)}^{a_{2}}e^{-\lambda {\left(\frac{1-e^{-x}}{1+e^{-x}}\right)}^{a_{1}}}e^{-\lambda {\left(\frac{1-e^{-x}}{1+e^{-x}}\right)}^{a_{2}}}dx\\ & =-\frac{e^{-\lambda }}{{\left(1-e^{-\lambda }\right)}^{2}}-\frac{2a_{1}}{{\left(1-e^{-\lambda }\right)}^{2}}\sum_{k=0}^{\infty }\frac{{\left(-\lambda \right)}^{k}}{k!}\int_{0}^{\infty }\frac{e^{-x}\;{\left(1-e^{-x}\right)}^{a_{1}+a_{2}k-1}}{{\left(1+e^{-x}\right)}^{a_{1}+a_{2}k+1}}e^{-\lambda {\left(\frac{1-e^{-x}}{1+e^{-x}}\right)}^{a_{1}}}dx\\ & +\frac{4a_{1}\lambda e^{-\lambda }}{{\left(1-e^{-\lambda }\right)}^{3}}\sum_{k=0}^{\infty }\frac{{\left(-\lambda \right)}^{k}}{k!}\int_{0}^{\infty }\frac{e^{-x}\;{\left(1-e^{-x}\right)}^{a_{1}+a_{2}k-1}}{{\left(1+e^{-x}\right)}^{a_{1}+a_{2}k+1}}e^{-\lambda {\left(\frac{1-e^{-x}}{1+e^{-x}}\right)}^{a_{1}}}dx\\ & +\frac{2a_{1}\lambda }{{\left(1-e^{-\lambda }\right)}^{2}}\sum_{k=0}^{\infty }\frac{{\left(-\lambda \right)}^{k}}{k!}\int_{0}^{\infty }\frac{e^{-x}\;{\left(1-e^{-x}\right)}^{2a_{1}+a_{2}k-1}}{{\left(1+e^{-x}\right)}^{2a_{1}+a_{2}k+1}}e^{-\lambda {\left(\frac{1-e^{-x}}{1+e^{-x}}\right)}^{a_{1}}}dx\\ & +\frac{2a_{1}\lambda }{{\left(1-e^{-\lambda }\right)}^{2}}\sum_{k=0}^{\infty }\frac{{\left(-\lambda \right)}^{k}}{k!}\int_{0}^{\infty }\frac{e^{-x}\;{\left(1-e^{-x}\right)}^{a_{1}+a_{2}\left(k+1\right)-1}}{{\left(1+e^{-x}\right)}^{a_{1}+a_{2}\left(k+1\right)+1}}e^{-\lambda {\left(\frac{1-e^{-x}}{1+e^{-x}}\right)}^{a_{1}}}dx\\ & =-\frac{e^{-\lambda }}{{\left(1-e^{-\lambda }\right)}^{2}}-\frac{2a_{1}}{{\left(1-e^{-\lambda }\right)}^{2}}\sum_{k=0}^{\infty }\frac{{\left(-\lambda \right)}^{k}}{k!}A^{*}\left(a_{1}+a_{2}k-1,a_{1}+a_{2}k-1,a_{1}\right)\\ & +\frac{4a_{1}\lambda e^{-\lambda }}{{\left(1-e^{-\lambda }\right)}^{3}}\sum_{k=0}^{\infty }\frac{{\left(-\lambda \right)}^{k}}{k!}A^{*}\left(a_{1}+a_{2}k-1,a_{1}+a_{2}k-1,a_{1}\right)\\ & +\frac{2a_{1}\lambda }{{\left(1-e^{-\lambda }\right)}^{2}}\sum_{k=0}^{\infty }\frac{{\left(-\lambda \right)}^{k}}{k!}A^{*}\left(2a_{1}+a_{2}k-1,2a_{1}+a_{2}k-1,a_{1}\right)\\ & +\frac{2a_{1}\lambda }{{\left(1-e^{-\lambda }\right)}^{2}}\sum_{k=0}^{\infty }\frac{{\left(-\lambda \right)}^{k}}{k!}A^{*}\left(a_{1}+a_{2}\left(k+1\right)-1,a_{1}+a_{2}\left(k+1\right)-1,a_{1}\right) \end{array}$$
